# A Dual pH‐ and Light‐Responsive Spiropyran‐Based Surfactant: Investigations on Its Switching Behavior and Remote Control over Emulsion Stability

**DOI:** 10.1002/anie.202114687

**Published:** 2022-03-23

**Authors:** Martin Reifarth, Marek Bekir, Alain M. Bapolisi, Evgenii Titov, Fabian Nußhardt, Julius Nowaczyk, Dmitry Grigoriev, Anjali Sharma, Peter Saalfrank, Svetlana Santer, Matthias Hartlieb, Alexander Böker

**Affiliations:** ^1^ University of Potsdam Institute of Chemistry Karl-Liebknecht-Straße 24–25 14476 Potsdam Germany; ^2^ Fraunhofer Institute for Applied Polymer Research (IAP) Geiselbergstraße 69 14476 Potsdam Germany; ^3^ University of Potsdam Institute of Physics and Astronomy Karl-Liebknecht-Straße 24–25 14476 Potsdam Germany

**Keywords:** Dual-Responsiveness, Manipulation of Emulsion Stability, Spiropyran Surfactant, Switchable Surfactants, pH-Dependent Photoresponsivity

## Abstract

A cationic surfactant containing a spiropyran unit is prepared exhibiting a dual‐responsive adjustability of its surface‐active characteristics. The switching mechanism of the system relies on the reversible conversion of the non‐ionic spiropyran (SP) to a zwitterionic merocyanine (MC) and can be controlled by adjusting the pH value and via light, resulting in a pH‐dependent photoactivity: While the compound possesses a pronounced difference in surface activity between both forms under acidic conditions, this behavior is suppressed at a neutral pH level. The underlying switching processes are investigated in detail, and a thermodynamic explanation based on a combination of theoretical and experimental results is provided. This complex stimuli‐responsive behavior enables remote‐control of colloidal systems. To demonstrate its applicability, the surfactant is utilized for the pH‐dependent manipulation of oil‐in‐water emulsions.

## Graphical abstract

A spiropyran‐based cationic surfactant is presented. Its architecture renders the molecule dual‐responsive, possessing thereby an unexpected switching behaviour: While photo‐stimulation influences the surface‐active behaviour of the compound under acidified conditions, no notable photo‐responsiveness is observed at pH‐values >6.5. This effect is demonstrated by manipulating the integrity of an oil‐in‐water emulsion.

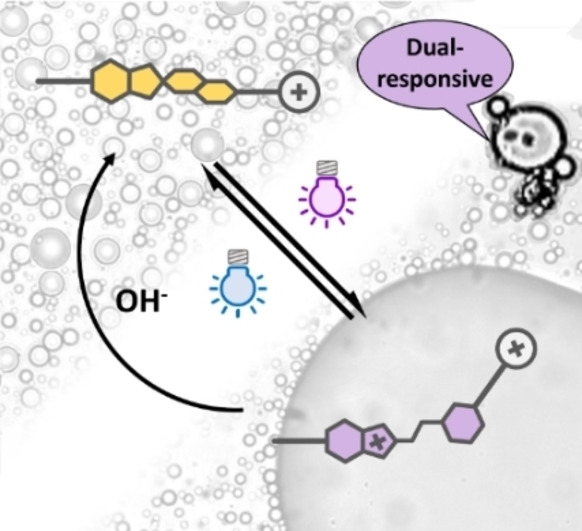

## Introduction

Stimuli‐responsive surface‐active molecules possess the ability to alter physico‐chemical characteristics, such as solubility, the tendency to form micelles^[1]^ or their adsorption behavior,[^2^, ^3^] upon the activation with an external trigger.[^1^, ^4^] This feature enables control, e.g. over the formation of vesicles and their manipulation[^5^, ^6^, ^7^] or surface tension of aqueous solutions,[^8^, ^9^, ^10^, ^11^] and can, therefore, be exploited to fabricate smart foams[^12^, ^13^] or emulsions with controllable stability[^14^, ^15^] among others. Switchable surfactants with a charged head group, beyond that, are suitable functionalizing agents. As switchable surfactants show an affinity to interact electrostatically with oppositely charged materials, they can be utilized to add a stimuli‐sensitive behavior to said materials, and can therefore induce a swelling behavior of microgels which exhibit ionic charges,[^16^, ^17^, ^18^] a conformational change in DNA as a naturally occurring polyelectrolyte,[^19^, ^20^, ^21^, ^22^] or cause an alteration of the topography of a surface grafted with polymer brushes.[^23^, ^24^]

Typical stimuli, hereby, are light,[^1^, ^4^] an external magnetic field,[^20^, ^25^] the pH value[^14^, ^15^, ^26^, ^27^, ^28^] or the concentration of gases in solution, such as carbon dioxide.[^29^, ^30^] As optical stimulation with an external light source is experimentally straightforward to implement, and since switching processes with light usually occur on a timescale of minutes,^[31]^ the rapid remote‐control of nano‐objects can conveniently be achieved using photo‐responsive surfactants.[^1^, ^4^] Other, yet considerable, systems respond on changes of the pH value, since they contain carboxylate^[14]^ and/or amino groups.[^14^, ^15^, ^26^, ^27^, ^28^] However, the manipulation of their surfactant properties demands a direct intervention, as an addition of acid or base to the system is necessary.[^14^, ^15^]

A combination of these pH and light responsivities could enable a more differentiated regulation of the properties of a colloidal system. A response of colloids used for drug delivery, for instance, facilitates an escape through the endosomal cellular barrier,^[32]^ or enables tumor tissue targeting.^[33]^ An additional light‐responsivity of the system could hereby add or enhance temporal and spatial control of cargo release.[^34^, ^35^] Providing a stimuli‐specific output as a result of a combination of different inputs, moreover, renders a dual‐responsive surfactant a suitable molecular logic gate,[^35^, ^36^, ^37^] which has gained interest in (bio‐) sensing and other applications.^[38]^

A suitable dual‐responsive moiety is spiropyran, which is able to reversibly ring‐open from its spiropyran (SP)‐ to the zwitterionic merocyanine (MC) form as a response to either pH‐ or photo‐activation.[^39^, ^40^] The peculiarity of this system lies in the tremendous difference in the solubility of both forms: The SP configuration possesses non‐polar properties, while its MC counterpart behaves either as a zwitter‐ or cation, depending on the external pH‐level. The concomitant different solubility behavior in water promises to translate into the surface‐activity of a respective surfactant.

Spiropyran‐based surfactants reported so far are restricted to architectures, in which the responsive unit represents the head group[^41^, ^42^] or is introduced as an additional terminus in the ω‐position of the molecule,[^43^, ^44^, ^45^, ^46^] including molecules with a low surface‐activity possessing a short alkyl linker (C_3_ to C_6_).[^11^, ^44^, ^46^] However, for surfactants based on azobenzene as light‐sensitive unit, it is known that an optimized remote control over their surface‐active behavior can be obtained, if the photoswitch is part of the hydrophobic tail of the surfactant.[^1^, ^4^, ^47^, ^48^] Therefore, we were motivated to adapt this geometry and to fabricate a stimuli‐responsive surfactant exhibiting a spiropyran functionality as part of its hydrophobic tail. Once synthesized, we characterize the pH‐ and light‐dependent surface‐active behavior of the surfactant and discuss the kinetics and thermodynamics of the switching process based on theoretical considerations. As a proof of concept, we demonstrate its exceptional multi‐responsive switching behavior with respect to its suitability to manipulate the stability of an oil‐in‐water emulsion.

## Results and Discussion

**Figure 1:**
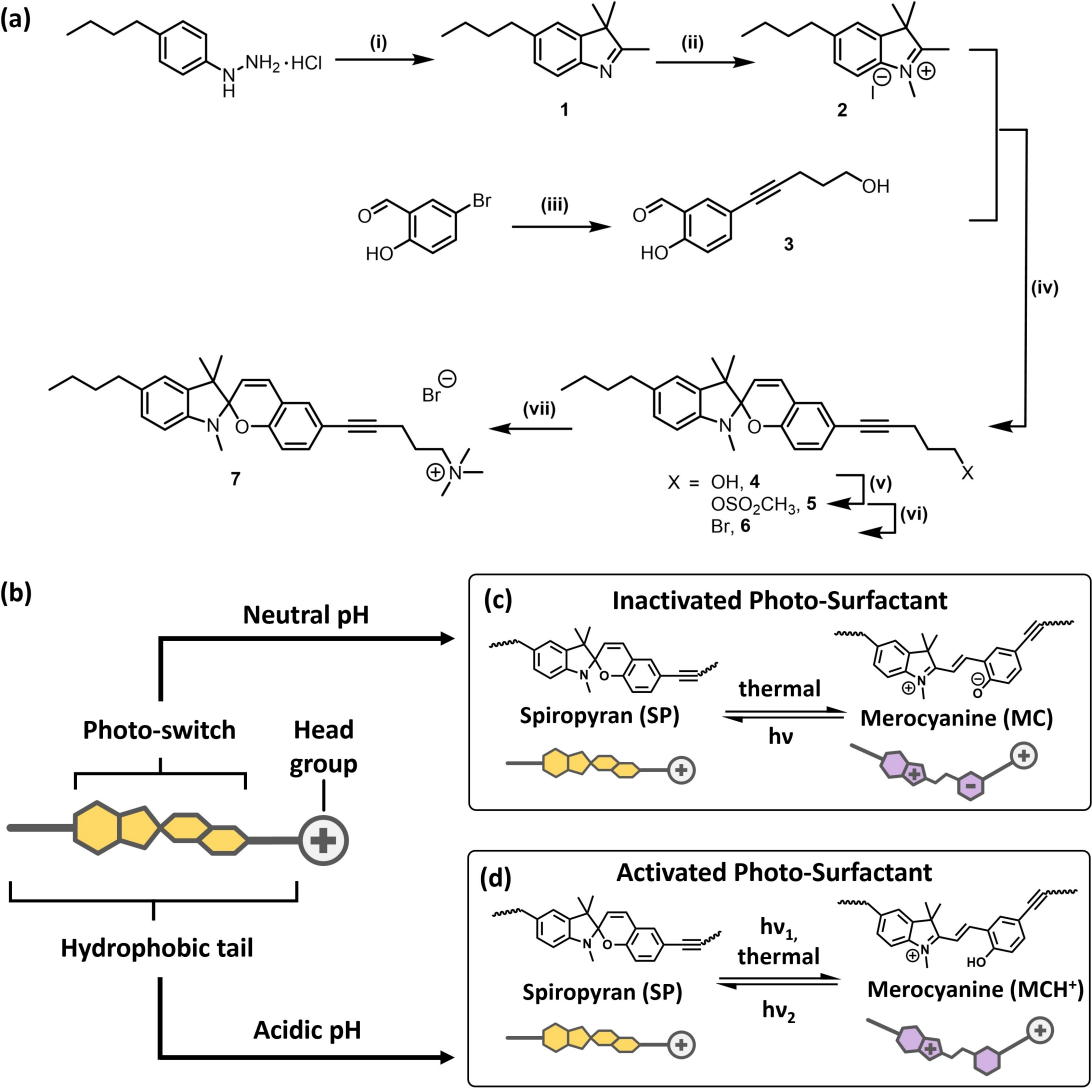
Synthetic route to the targeted structure (a), reaction conditions: i) 3‐methylbutan‐2‐one, acetic acid, reflux, 12 h, 74 %, ii) methyl iodide, acetonitrile, rf, 21 h, 95 %, iii) pent‐4‐yn‐1‐ol, NEt_3_, Pd(PPh_3_)_4_, CuI, 80 °C, 16 h, 78 %, iv) 1. NaOH, H_2_O, 2. EtOH, rt, 5 h, 80 %, v) CH_3_SO_2_Cl, NEt_3_, CH_2_Cl_2_, room temperature, 22 h and vi) 1‐butyl‐3‐methylimidazolium bromide, 60 °C, 2 h, 53 % over two steps, vii) N(CH_3_)_3_, EtOH, 50 °C, 4 d, 88 %. Scheme of dual‐responsive surfactant (b), photo‐switching of **7** at neutral pH (c) and acidic pH (d).

The surface‐active compound discussed in this study combines the classical architecture of a surfactant with a dual photo‐ and pH‐responsive unit. The multistep fabrication of this molecule is outlined in Figure 1a. The resulting structure consists of a charged head group, an alkyl spacer connecting the head with the switchable spiropyran unit, as well as a terminal alkyl chain as depicted in Figure 1b, representing a structural architecture adapted from the optimized molecular geometry of azobenzene‐based surfactants.[^1^, ^48^] In this structure, the spiropyran moiety is incorporated into the hydrophobic tail of the surfactant and thus differs from previously reported structures,[^43^, ^44^, ^45^, ^46^] in which the spiropyran unit is substituted via its indole‐nitrogen. Due to ring‐opening of the spiropyran (SP) moiety within the hydrophobic tail—yielding the corresponding merocyanine form, which is zwitterionic (MC) under neutral and positively charged (MCH^+^) under acidic conditions—the molecule is expected to alter its solubility behavior in water (Figure 1c, d). Experimental details (Figure S1–S7) and nuclear magnetic resonance (NMR) spectra (Figure S8–S23) of all products are presented in the Supporting Information.

Starting from the commercially available 4‐(*n*‐butyl)phenylhydrazine, 5‐butyl‐2,3,3‐trimethyl‐3H‐indole (compound **1**) was synthesized following the classical *Fischer* indole synthesis route. Compound **2** was obtained in an excellent yield (95 %) by methylation using methyl iodide. Simultaneously, the salicylaldehyde derivative **3** was attained via a *Sonogashira*‐type cross‐coupling, which has been reported to be an established route for the functionalization of spiropyrans.[^49^, ^50^] After a deprotonation of **2** with sodium hydroxide (monitored by ^1^H NMR, Figure S12), it was reacted with **3** to yield the spiropyran‐functionalized alcohol **4**. The conversion of the alcoholic hydroxy functionality to the cationic ammonium group was conducted in three steps, starting with the introduction of the mesylate group to yield **5** and the subsequent bromination to **6** using an ionic liquid,^[51]^ followed by the successive quarternization with trimethylamine to yield the cationic surfactant **7** (purity proven by electrospray‐mass spectrum, see Figure S24).

**Figure 2:**
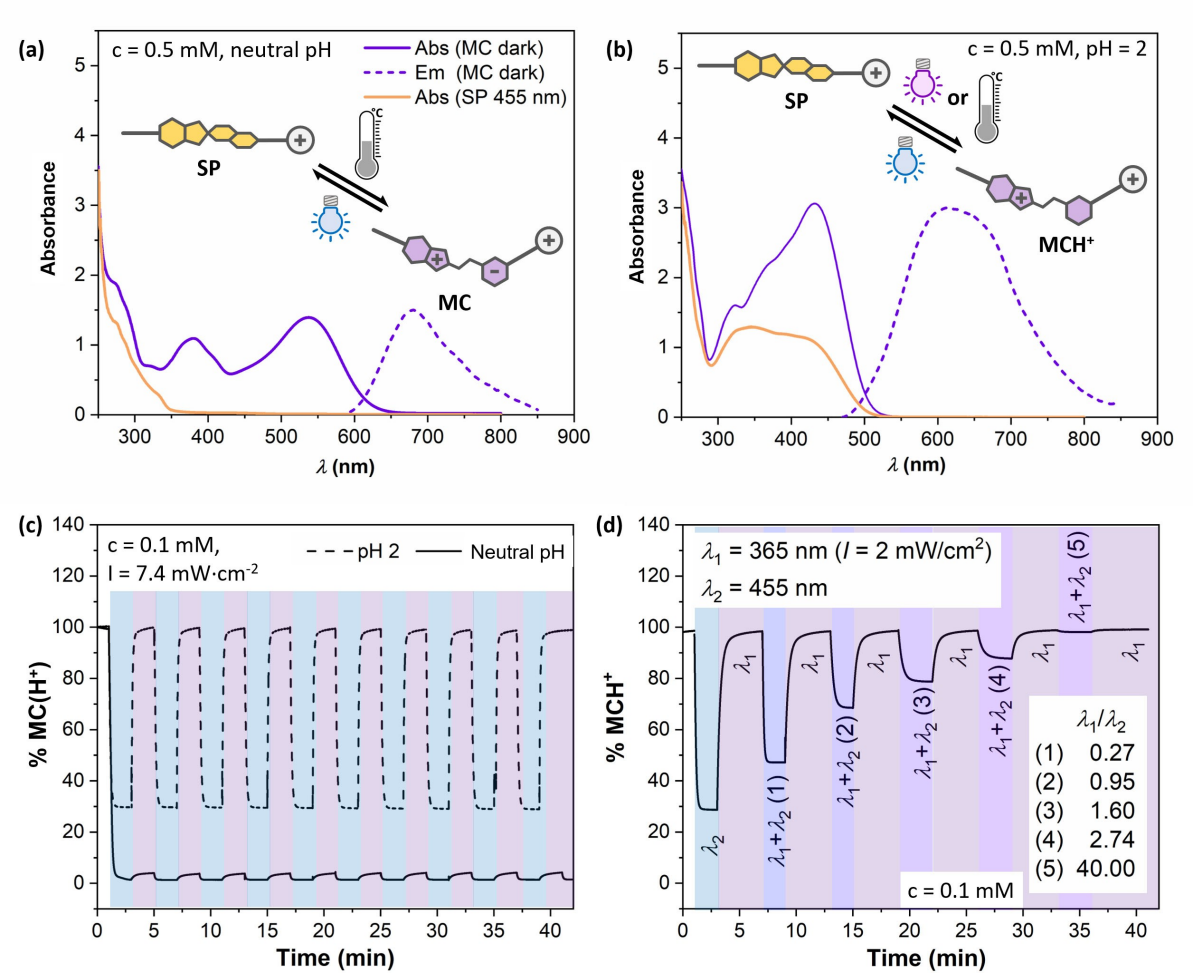
Photochemical characteristics and photostability of the photo‐switchable surfactant **7**. a) Absorption and emission spectra of **7** (*c*=0.5 M) at neutral pH. b) Absorption and emission spectra of **7** at acidic conditions (*c*=0.5 mM, 3 equivalents CF_3_COOH). Note, that the absorption spectrum (orange line) likely represents a sum of SP and residual MCH^+^ as a result of non‐complete switching). c) Reversibility and photo‐stability of the switch at neutral and acidic pH. d) Control of [SP]:[MCH^+^] ratio via illumination at two wavelengths (λ_1_=365 nm and λ_2_=455 nm) at varying intensity ratios.

Obtained as a light‐violet solid, compound **7** dissolves readily in milliQ water. The resulting faint yellow solution turns red and intensifies its color over time, indicating that the merocyanine (MC) form of the surfactant is thermodynamically stable in water (Figure 2a). Showing additionally fluorescent properties, MC can be photo‐activated, e.g. by irradiation with visible light at 455 nm, to be converted into the SP form with a half‐life of ≈1 min 24 s.

Emission spectra of light sources used for irradiation are shown in Figure S25 and time‐dependent measurements are shown in Figure S26. The SP form, being thermodynamically instable, relaxes back to the MC form with a half‐life of approx. 6 min 12 s (Table S1). UV/Vis absorption and fluorescence spectra are provided in Figure 2a. Under acidic conditions, the solution possesses an orange appearance, which was analyzed via UV/Vis spectroscopy as well (Figure 2b). The absorption band shows a hypsochromic shift in comparison to the MC form which is indicative for its protonation to yield the MCH^+^ form. Constituting a doubly positively charged molecule with a cationic head group and another positive charge along its tail (Figure 2b), this thermodynamically stable MCH^+^ can be activated by photo‐irradiation with 455 nm (half‐life of ≈52 s). The resulting SP form relaxes back to MCH^+^ with a thermal half‐life of 37.5 min at ambient temperature. Interestingly, this process can be induced by photo‐stimulation as well, whereby an irradiation with 365 nm results in the quick formation of MCH^+^ (half‐life ≈38 s). This is quite remarkable, since the photo‐controllability of spiropyran switching is reported to be possible only into one direction.^[39]^ Section 3.1 in the Supporting Information (Figures S26 to S28 as well as Table S1) provides a more detailed discussion on the kinetics.

To demonstrate the photo‐switching of the surfactant in more detail, two defined wavelengths (365 and 455 nm) were applied in an alternating manner. Figure 2c reveals, that the acidified surfactant can be subjected to at least 10 switching cycles without forfeiting notable stability, whereby a distinct switching between MCH^+^ and SP can be achieved. Under neutral conditions, however, the reversibility of photo‐switching is limited, since irradiation with UV light (365 nm) only results in the conversion of 8 % SP to MC (Figure 2c). Due to the good photo‐controllability of the transition under acidic conditions, the simultaneous irradiation with the respective wavelengths (365 and 455 nm) allows to control the [SP]:[MCH^+^] ratio (Figure 2d). Interestingly, the forward as well as the back reaction exhibit similar half‐lives <1 min (Table S1), which means that the molar ratios of both isomers can conveniently be controlled by the intensity ratio of the used irradiation lamps (Figure 2d).

**Figure 3:**
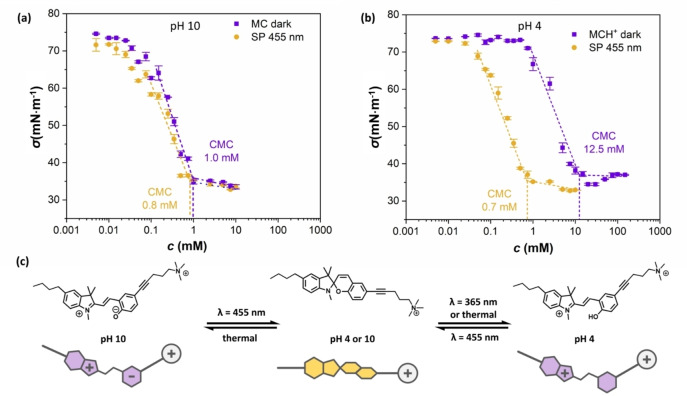
Dependence of the surface energy on the surfactant concentration for alkaline (a) and acidic (b) pH level at two irradiation conditions as indicated in the legend. The corresponding CMC at different pH and irradiation (blue colour indicates MC/MCH^+^ forms, yellow depicts SP state) are depicted on the plots. c) The difference in behaviour can be explained by the protonation of MC to MCH^+^.

To quantify its pH‐dependent behavior, compound **7** was acidified and subsequently titrated with NaOH in its MC (relaxed) as well as its SP (photo‐activated) configuration. The respective titration curves (potentiometric curve in Figure S29, UV/Vis detection in Figure S30) indicate a transformation of MC to MCH^+^ under thermodynamically relaxed conditions, which is characterized by a p*K*_a_ value of 6.4. In contrast, the absence of a half‐equivalence point in the titration curve of the activated molecule suggests, that the SP form does not undergo a protonation reaction within the tested pH range. A more detailed discussion is provided in section 3.2 in the Supporting Information. To characterize compound **7** regarding its dual‐responsive surface‐active behavior, the concentration‐dependent surface tension of its aqueous solutions was determined. In detail, pendant‐drop measurements were performed to characterize the surfactant in an alkaline (Figure 3a) and acidic (Figure 3b) solution under both photo‐activated and relaxed conditions regarding the critical micelle concentrations (CMCs) of respective forms. In alkaline solution, CMCs of 0.8 mM (SP form) and 1.0 mM (MC form) were detected, whereas at acidic conditions, CMC values of 0.7 mM (SP form) and 12.5 mM (MC form) were found. Hence, the photo‐mediated switching can be translated into a change in surface activity at acidic pH, but has little influence under alkaline (and neutral, Figure S31) conditions.

The significant CMC difference in an acidic environment can easily be explained by the fact that switching from SP to MCH^+^ transfers compound **7**, initially possessing a classical surfactant architecture into a doubly charged molecule (Figure 3c) with enhanced solubility and reduced surface‐activity. The insignificant CMC difference under alkaline conditions, however, appears, at a first glance, surprising. Since the presence of a zwitterionic moiety in the open MC form promises a likewise improved solubility, the switching process should be accompanied by a substantial reduction of its surface‐active behavior.

This unexpected effect is best explained by the ability of MC to switch back to SP when aggregating into a micelle. Since the micellization is a thermodynamically favored process driven by an entropy gain from released solvent molecules (e.g. for cetyltrimethylammonium bromide, CTAB, providing an energy gain of ≈23 kJ mol^−1^ at room temperature),^[52]^ we hypothesize that for our system the gain of the free energy for SP micellization is even larger, as water molecules solvating the zwitterionic structure of the solubilized MC form are released during the ring‐closure reaction. Thus, a low difference of the Gibbs free energies between SP and MC at neutral or alkaline pH would explain the observed behavior.

**Figure 4:**
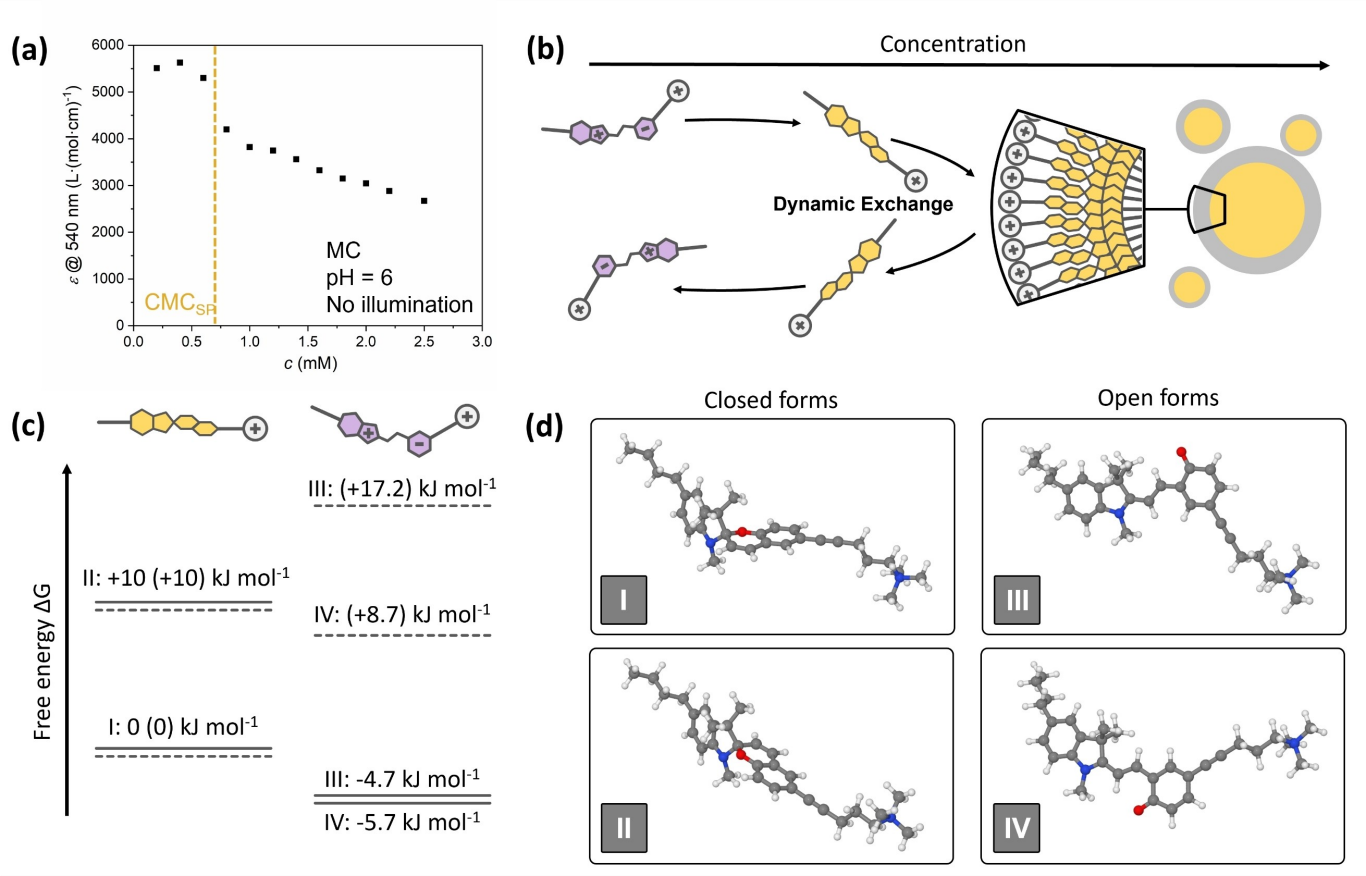
Explanation of the switching behaviour of the surfactant under conditions pH >6.4. a) Determination of the molar extinction coefficient *ϵ* at a wavelength of 540 nm in dependence on the concentration. At concentrations>CMC, the extinction coefficient decreases as a result of the partial backswitching from MC to SP. b) Schematic representation of the influence of concentration on the state of the surfactant. c) Energy states (relative Gibbs free energies) of **7**, as calculated by DFT using the B3LYP+D3(BJ)/def2‐TZVP/PCM (water) level (solid lines), and gas‐phase B3LYP+D3(BJ)/def2‐TZVP calculations (dotted lines, numbers in parentheses); left: closed‐ring form, right: open‐ring form. d) Corresponding geometry‐optimized structures at the B3LYP+D3(BJ)/def2‐TZVP/PCM(water) level.

To substantiate this hypothesis, we recorded UV/Vis absorption spectra of differently concentrated solutions of the surfactant (Figure S32), and plotted the molar extinction coefficients ϵ at λ=540 nm (being characteristic for the absorption of the MC form with ϵ_SP_(540 nm)≈0) against the surfactant concentration (Figure 4a). For concentrations>CMC of SP (0.8 mM), a decrease of the value of ϵ is observed. This decrease, despite increasing concentrations, is explained by a relative removal of MC species from the equilibrium for the benefit of SP concentration, which is well in accordance with the hypothesized mechanism (Figure 4b). The consequent opposite, i.e. the intensification of the red color of a surfactant solution upon further dilution with water, is demonstrated in the supplementary video V1 (discussion in section 4 of the Supporting Information). Theoretical calculations were performed to underline this explanation under thermodynamic aspects. For this purpose, energy states of the geometry‐optimized relevant molecular configurations were assessed. During geometry optimizations, different species (minima on the potential energy surface) are found for both the open and closed forms. Note, that a more detailed discussion can be found in section 5 of the Supporting Information. In particular, i) closed‐form structures I and II, differing by the spatial arrangement of the *O*‐atom with respect to the *N*‐containing ring, and ii) open‐form structures III and IV, differing by rotation of the O‐containing ring around the adjacent CC bond (see Figure 4d), are found. Gibbs free energies of these isomers, based on density functional theory (DFT) calculations, executed with a polarizable continuum model (PCM) to account for solvent effects, are shown in Figure 4c. In agreement with experimental observations, these calculations predict that the MC form is thermodynamically stable in water. Compared to the SP form (referenced to 0 kJ mol^−1^ for structure I and 10 kJ mol^−1^ for structure II, Figure 4c, d), Δ*G* for MC was determined to be −4.7 kJ mol^−1^ or −5.7 kJ mol^−1^ (structure III or IV, respectively, Figure 4c, d). Due to the fact that the energy difference between SP and MC with <5.7 kJ mol^−1^ is rather low in a pH‐neutral or alkaline case, a conversion from MC to SP might be possible (energy diagram with transition states are shown in Figure S33). Being in a relevant energetic range of the entropy gain—since i) water molecules hydrating the ionic parts of the MC molecule are released and ii) water molecules dissolving the surfactant are released during the molecule's assembly to a micelle—the aggregation of SP into micelles, therefore, represents the driving force for switching at concentrations above the CMC. Note, that the optical behavior shown in Figure 4a may alternatively be explained by the formation of molecular excitons as described for azobenzene surfactants;[^53^, ^54^] the theoretical considerations, however, make the aforementioned explanation plausible. In contrast, the free energy difference between both states is much more pronounced under acidic conditions (see details in the Supporting Information, Figure S34 and accompanying discussion in section 5) and, moreover, spiropyrans were reported to undergo a spontaneous ring‐opening upon protonation.^[55]^ These findings support our initial hypothesis about the unique behavior of the surfactant presented herein. It should be noted, that for comparison all calculations were also performed under gas‐phase conditions (results in brackets in Figure 4). These calculations suggest an enhanced thermodynamic stability of the SP form over the MC one and confirm the literature‐known fact, that the thermodynamic balance is sensitive to the micro‐environment.^[39]^

**Figure 5:**
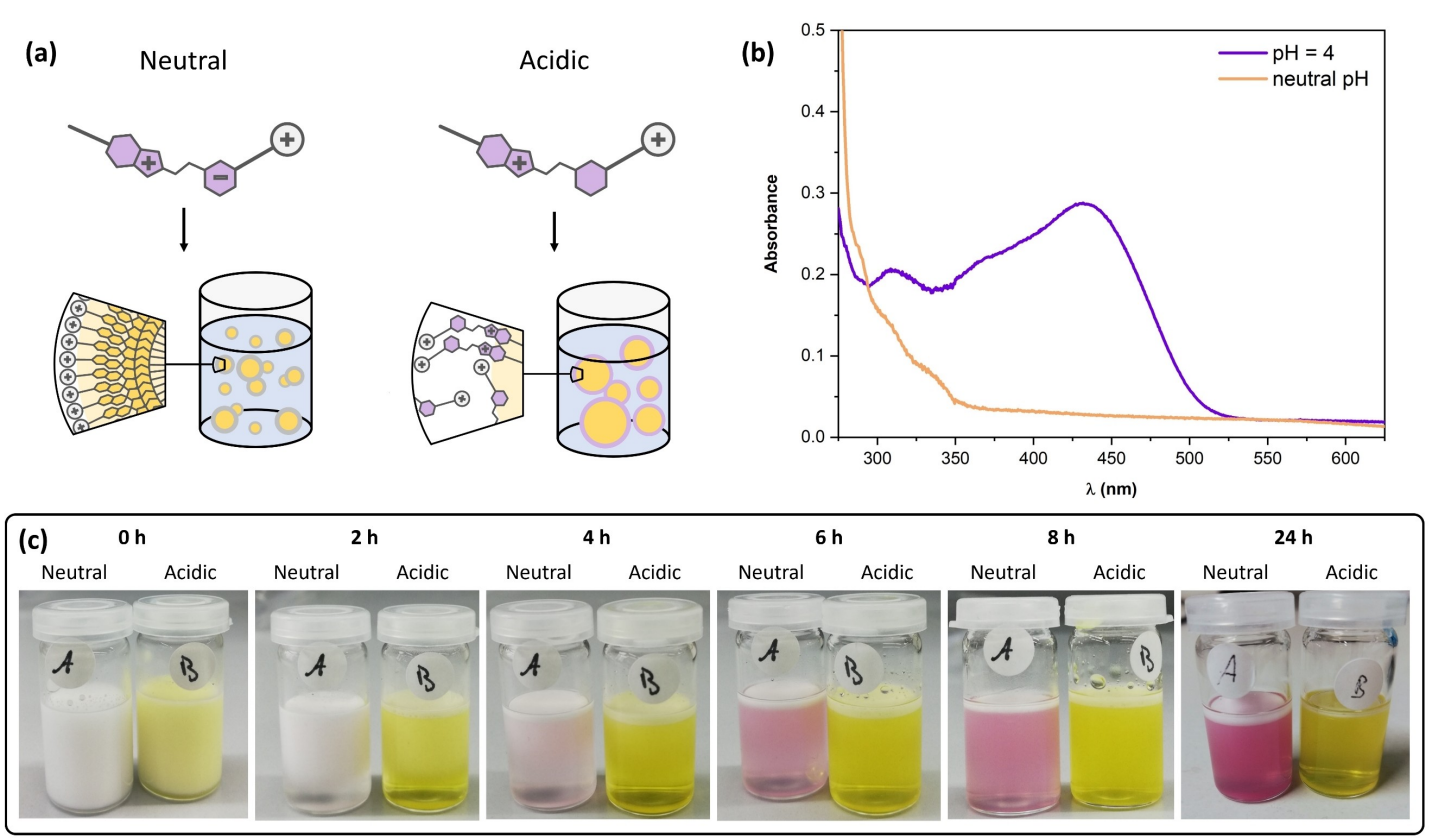
Behaviour of compound **7** in its thermally relaxed state at an oil/water interface studied on a toluene/water oil‐in‐water emulsion. a) Schematic representation of the suspected assembly. In a neutral emulsion, the compound is expected to be present in its SP form. In an acidic environment, the simultaneous presence of SP and MCH^+^ is likely, as indicated by the UV/Vis absorption characteristics shown in (b). The absorption spectra were measured against an emulsion stabilized with CTAB. c) Photographs of the emulsions after different times.

Having investigated the behavior of compound **7** in aqueous media, we were next interested in its capability to assemble at an oil/water interface. For this purpose, we emulsified toluene as oil phase in an aqueous medium, using the surfactant (c<CMC) in its thermally relaxed MC(H^+^) form under both, acidic and neutral pH value (Figure 5a). The UV/Vis absorption spectra of the resulting oil‐in‐water emulsions (Figure 5b) provide an insight into the switching state of the surfactant molecules at the interface. It should be noted, that we show baseline‐corrected spectra, using a CTAB‐stabilized emulsion as a reference, to compensate absorption loss due to scattering. The absorption spectrum of the pH‐neutral emulsion is in good accordance with the UV/Vis characteristics for SP, indicating that the deployed MC molecule undergoes spontaneous switching during the emulsification. We consider this switching an entropy‐driven assembly process, during which the SP molecules align at the oil‐water‐interface, where they stabilize the emulsion droplets.

At the interface, the SP form is thermodynamically stabilized in the neutral case. Under acidic conditions, however, the UV/Vis absorption appears to be an overlap of SP and MCH^+^ characteristics, pointing towards the simultaneous presence of both forms. These findings can explain the different emulsion integrities over time: Whereas the pH‐neutral emulsion exhibits a reasonable stability over 24 h, the acidic mixture demulsifies over that time period, as photographs (Figure 5c) along with the corresponding transmission light micrographs (Figure S35) suggest.

**Figure 6:**
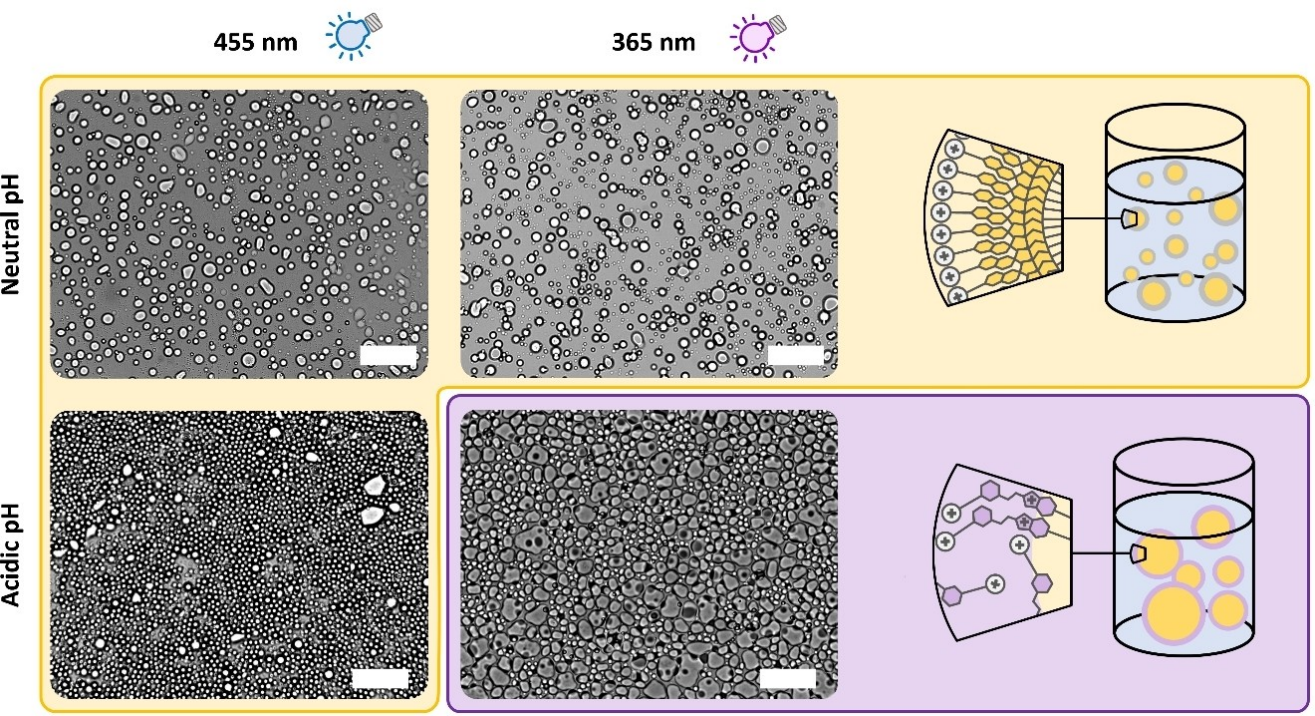
Irradiation of emulsions with light. Emulsions with different pH values were irradiated with UV and blue light for 10 minutes. Irradiation with blue light keeps the emulsion stable. Under UV light, however, the acidic emulsion destabilizes, while the neutral one stays intact. The scale bars in the transmission light micrographs are 100 μm.

Motivated by the difference in emulsion stabilities, we were interested, whether these switching characteristics can be exploited for the targeted destabilization of an emulsion. Accordingly, toluene oil‐in‐water emulsions were prepared, using compound **7** in its surface‐active SP form (requires previous irradiation with 455 nm) as an additive to CTAB for stabilization. Subsequently, the emulsions—prepared under pH‐neutral and acidic conditions—were exposed to irradiation with UV‐ as well as blue light (Figure 6, supplementary videos V2–V5). As expected, irradiation with blue light did not affect the integrity of the emulsion droplets notably (supporting video V2 and V4, characteristic frames in Figure 6), since photons with this wavelength photochemically stabilize the surface‐active SP form. Exposure with UV light, however, provides a more differentiated image over the emulsion stability: Whereas the pH‐neutral emulsion remained unaffected (supporting video V3), the oil‐in‐water biphasic system could be demulsified under acidic conditions (supporting video V5).

This behavior can be explained by the photochemical activity of the SP form at the oil/water interface in different pH‐regimes. While UV light is incapable to switch SP to MC under neutral conditions, it was shown to stimulate the conversion of the SP to the less surface‐active MCH^+^ form at low pH values, which eventually leads to demulsification.

Figure 6 summarizes the respective results, showing that only a combination of an acidic pH‐value and irradiation with UV light induces demulsification, whereas the compliance with only one or even none of the appropriate conditions keeps the emulsion stable. Compound **7** can thus be considered a molecular logic ‘AND’ gate, transferring the two different inputs, pH value and light, into a drop of the surface‐activity as a selective output.^[38]^ Even though molecular logics of spiropyrans have been discussed in previous studies, using e.g. fluorescent,[^56^, ^57^] UV/Vis absorptive,[^58^, ^59^] or chiroptical[^60^, ^61^] signals as outputs, their implementation in the context of emulsion stability is not reported. The peculiar pH‐dependent energy differences of the photoisomerization process, which are a consequence of the substitution pattern of the spiropyran moiety along with its position in the molecule, render compound **7** unsensitive to neutral, but photo‐sensitive to acidic pH values. From this point of view, it differs from alternative switchable surfactants, which possess merely photo‐sensitive moieties, such as azobenzene[^1^, ^4^] or ‐pyrazole^[11]^ in its backbone, as well as spiropyran‐based systems, into which the photoactive unit is introduced as the head group.[^43^, ^44^, ^45^, ^46^]

Its differentiated dual‐responsive behavior encouraged us to employ compound **7** as an auxiliary for the control of a reaction in a biphasic system. We deem this process a dedicated application for our substance, since it relies on the selective control of the integrity of an emulsion: A stable emulsion is required during the reaction process to enable an efficient interaction between the reaction partners in both phases, while the extraction of the products after the completion of the reaction demands its targeted destabilization. Compound **7** can be highly valuable in this context, as the molecular logics inherent to this compound can elegantly be employed to assess the reaction progress. For this purpose, a biphasic reaction was executed using the toluene emulsion droplets as microreactor, during which a constant release of acidic components into the water phase occurs. A UV‐triggered emulsion destabilization is only achieved under acidic conditions and, the demulsification is facilitated merely after a decent conversion of the starting materials.

**Figure 7:**
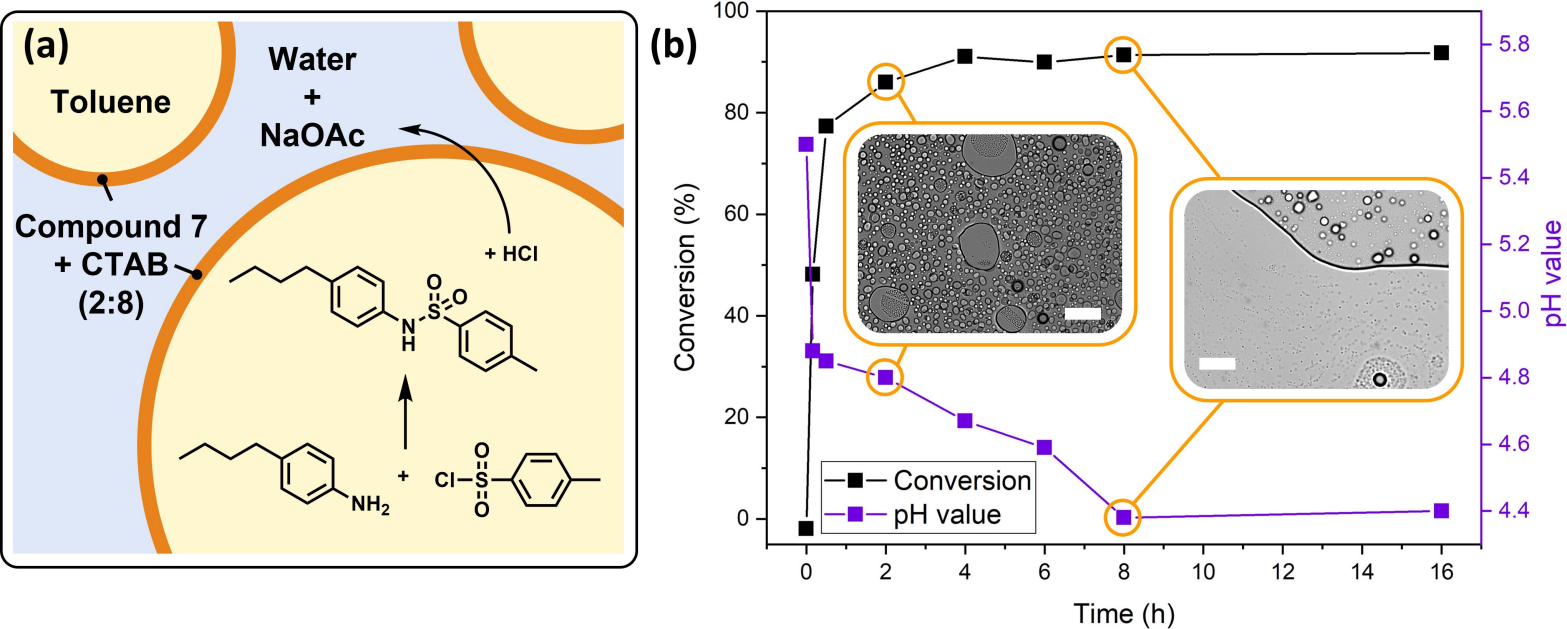
Overview over the chemical reaction conducted in the emulsion droplets. a) Schematic overview. The two organo‐soluble reaction partners are present in the toluene phase. During the reaction process, acidic compounds are released into the aqueous continuous phase. b) Overview over the time evolution of the reaction. As monitored, the pH value drops over the time. Simultaneously, the conversion of 4‐(*n*‐butyl)aniline increases as monitored by gas chromatography. An emulsion could not be destabilized after 2 h reaction time, whereas after 8 h, UV irradiation severely affected the emulsion integrity. The insets in (b) are respective transmission light micrographs of the emulsions after irradiation with UV light for 10 min (2 h) or 9 min (8 h). Scale bars are 50 μm.

For this purpose, we conducted the Schotten–Baumann‐type amide formation between 4‐(*n*‐butyl)aniline with 4‐toluenesulfonyl chloride. The reaction partners, dissolved in toluene, were emulsified with an aqueous sodium acetate buffer as schematically depicted in Figure 7a. Figure 7b reveals an insight into the reaction kinetics. Based on the determination of the pH value and gas chromatography (detailed insight in Figure S36), a maximum conversion of approx. 90 % is reached after 4 h, and after 8 h, a minimum pH value was measured. The emulsion was sampled, and aliquots were irradiated with UV light. As expected from the results presented in Figure 6, the emulsion could not significantly be destabilized after 2 h, as the pH value is not sufficiently low after that reaction time (Figure 7b, supplementary video V6). With a further conversion of the starting material after 8 h, however, UV‐stimulation led to notable demulsification (supplementary video V7). Note, that 100 % conversion could not be achieved, which was attributed to a limited stability of 4‐toluenesulfonyl chloride in an aqueous emulsion setup. The respective kinetic investigations are demonstrated in Figure S37 and in the supplementary video V8.

## Conclusion

In summary, we present the preparation of a dual pH‐ and photo‐responsive surfactant bearing a spiropyran moiety in its hydrophobic backbone. The surfactant is analyzed regarding its photophysical properties and probed with respect to its micellization behavior. These investigations show a pronounced difference of the CMC in an acidic environment, whereas this behavior is suppressed at a pH above 6.4. Plausible theoretical considerations are provided, and the underlying switching mechanisms are investigated experientially regarding their kinetics. Eventually, the capability of the compound to manipulate oil‐in‐water emulsions stabilities via light and pH value is examined. As an application, we implement our surfactant as a stabilizer for an emulsion used for reaction control. Such a dual‐responsive behavior can be exploited in the development of smart colloidal materials, able to differentiate between various micro‐environments and can be anticipated to be applied, e.g. in drug delivery applications or in molecular logics among others.

## Conflict of interest

The authors declare no conflict of interest.

1

## Supporting information

As a service to our authors and readers, this journal provides supporting information supplied by the authors. Such materials are peer reviewed and may be re‐organized for online delivery, but are not copy‐edited or typeset. Technical support issues arising from supporting information (other than missing files) should be addressed to the authors.

Supporting Information

Supporting Information

Supporting Information

Supporting Information

Supporting Information

Supporting Information

Supporting Information

Supporting Information

Supporting Information

## Data Availability

The data that support the findings of this study are available in the supplementary material of this article.
