# Supplementary material for: A Dual pH‐ and Light‐Responsive Spiropyran‐Based Surfactant: Investigations on Its Switching Behavior and Remote Control over Emulsion Stability
Source: Angew Chem Int Ed Engl. 2022 Mar 23;61(21):e202114687. doi: 10.1002/anie.202114687 (PMC9400902; doi:10.1002/anie.202114687)
Supplement: Supplementary file 1 — Supporting Information [file ANIE-61-0-s003.pdf]

Angewandte Chemie
Eine Zeitschrift der Gesellschaft Deutscher Chemiker

Supporting Information

A Dual pH- and Light-Responsive Spiropyran-Based Surfactant: Investigations on Its Switching Behavior and Remote Control over Emulsion Stability

M. Reifarth, M. Bekir, A. M. Bapolisi, E. Titov, F. Nußhardt, J. Nowaczyk, D. Grigoriev, A. Sharma, P. Saalfrank, S. Santer, M. Hartlieb, A. Böker**

Table of Contents

Table of Contents 2
1. Experimental Section 3
1.1 Chemical Syntheses 3
1.1.1 Synthesis of 5-*n*-butyl-2,3,3-trimethyl-3*H*-indole (1) 4
1.1.2 Synthesis of 5-butyl-2,3,3-trimethyl-3*H*-indol-1-ium iodide (2) 5
1.1.3 Synthesis of (4-Hydroxy-5-formylphenyl)-pent-4-yne-1-ol (3) 5
1.1.4 5-(5'-*n*-butyl-1',3',3'-trimethylspiro[chromene-2,2'-indolin]-6-yl)pent-4-yn-1-ol (4) 6
1.1.5 5-(5'-butyl-1',3',3'-trimethylspiro[chromene-2,2'-indolin]-6-yl)pent-4-yn-1-ol methyl sulfonic acid ester (5) 8
1.1.6 5-(5'-butyl-1',3',3'-trimethylspiro[chromene-2,2'-indolin]-6-yl)pent-4-yn-1-bromide (6) 9
1.1.7 5-(5'-butyl-1',3',3'-trimethylspiro[chromene-2,2'-indolin]-6-yl)pent-4-yn-1-(trimethyl-ammonium) bromide (7) 10
1.2 Photophysical characterization of the system 11
1.3 Potentiometric titration of compound 7 12
1.4 Determination of the critical micelle concentration 13
1.5 Computational details 13
1.6 Emulsification experiments 14
1.6.1 Photophysical characterization of the emulsions 14
1.6.2 Photo-manipulation of the emulsions 15
1.6.3 Reaction control using emulsion droplets as microreactors 16
1.6.4 Targeted destabilization of the emulsion droplets used as microreactors 17
2. Supplementary spectra characterizing the target compound and its intermediates 19
3. Photophysical investigations 28
3.1 Kinetic Investigations 28
3.2 Determination of the pH-dependent behavior of the surfactant 34
4. Micellization behavior of the surfactant 36
5. Theoretical considerations 37
6. Emulsification experiments 40

1. Experimental Section

1.1 Chemical Syntheses

Unless stated otherwise, all chemicals were used as obtained from the supplier without further purification. 4-(*n*-butyl)phenylhydrazine-hydrochloride (>95%) was obtained from ABCR. 3-Methylbutan-2-one (>98%), and 5-bromosalicylaldehyde (>98%) were purchased from Alfa Aesar. Methyl iodide (>99.5%), methanesulfonyl chloride (>99.7%), 1-Butyl-3-methylimidazolium bromide (>97%), trimethylamine (31 to 35 wt.-% in ethanol, stabilized with toluene), and triethylamine (>99%) were obtained from Sigma Aldrich. Pent-4-yne-1-ol (>96%) and tetrakis(triphenylphosphino) palladium(0) (>97%) was purchased from TCI. Copper(I) iodide was purchased from Merck. Silica gel 60 (0.04-0.063 mm, 400-230 mesh) for chromatography as well as dichloromethane (>99.5%, for synthesis) were obtained from Carl Roth. Sodium hydroxide (NaOH, >99.8%), diethyl ether (>99.5%), ethanol (96%), *n*-hexane (>95%), sodium sulfate (Na_2SO_4, >99%, ACS reagent), and glacial acetic acid (99.5%) were obtained from Chemsolute. Ethyl acetate (Hypersolv, for HPLC), acetonitrile (Hypersolv, for HPLC), tetrahydrofurane (Hypersolv, for HPLC), as well as *n*-heptane (Normapur) were purchased from VWR. Sodium carbonate (Na_2CO_3, >99.7%) was obtained from Sigma Aldrich. Deuterated chloroform ($CDCl_3$, 99.8 atom-% D) was purchased from Sigma Aldrich. NMR measurements were recorded on a Bruker 400 MHz spectrometer. High-resolution mass spectra were recorded by electron impact time-of-flight (EI-ToF) or electrospray time-of-flight (ESI-ToF) using a Waters Micromass Manchester instrument. ESI data presented in Figure S 24 was recorded with a PerkinElmer Flexar SQ 300 MS Detector (using the PerkinElmer V2.2 Software for data analysis).

1.1.1 Synthesis of 5-*n*-butyl-2,3,3-trimethyl-3*H*-indole (1)

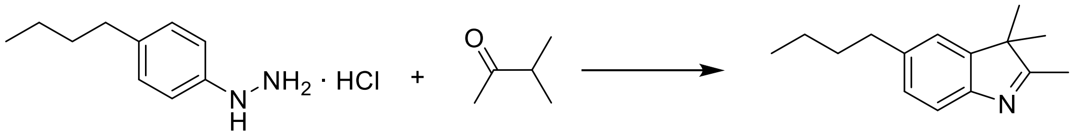

Figure S 1: Schematic representation of the synthesis of compound **1**.

In a round-bottom flask, 4-(*n*-butyl)phenylhydrazine-hydrochloride (3.00 g, 15 mmol) was weighted in and suspended in 37 mL glacial acetic acid. To this solution, 3-methylbutan-2-one (1.55 g, 18 mmol) was added dropwise. Thereafter, the suspension was refluxed in a N_2 atmosphere, whereupon the 4-(*n*-butyl)phenylhydrazine-hydrochloride dissolved completely to yield a light-pink solution. After 12 h heating under reflux, the solution was cooled to room temperature. The red-violet solution was, next, diluted with water, whereby a precipitate formed. Extraction of this suspension with CH_2Cl_2 and further washing of the organic phase with a 1M Na_2CO_3 solution provided a clear organic phase. This phase was dried over Na_2SO_4 and evaporated to dryness to yield a dark liquid. Column chromatography (SiO_2, *n*-hexane/ethyl acetate = 3:1) provided the pure compound **1** as a yellowish oil (2.38 g, 74 % yield). ^{1}H-NMR ($CDCl_3$, 400 MHz): δ 7.44 (Ar-H, d, J = 7.72 Hz, 1H), 7.12 (Ar-H, q, J = 3.83 Hz, 2H), 2.66 (Ar-$C\underline{H}_2$-CH_2-CH_2-CH_3, t, J = 7.81 Hz, 2H), 2.28 (Indol-CH_3, s, 3H), 1.63 (Ar-CH_2-$C\underline{H}_2$-CH_2-CH_3, m, 2H), 1.39 (Ar-CH_2-CH_2-$C\underline{H}_2$-CH_3, quart., J = 7.40 Hz, 2H), 1.31 (Indol-$(CH_3)_2$, s, 6H), 0.96 (Ar-CH_2-CH_2-CH_2-$C\underline{H}_3$, t, J = 7.34 Hz, 3H). ^{13}C-NMR ($CDCl_3$, 100 MHz): δ 187.0, 151.6, 145.7, 140.1, 127.5, 121.4, 119.4, 53.7, 35.7, 34.1, 23.3, 22.5, 15.3, 14.0. HRMS (EI, m/z): 215.1674 (calc. for $[M]^+$ $C_{15}H_{21}N$), found: 215.1683 (Δm/z = + 4.3 ppm).

1.1.2 Synthesis of 5-butyl-2,3,3-trimethyl-3*H*-indol-1-ium iodide (2)

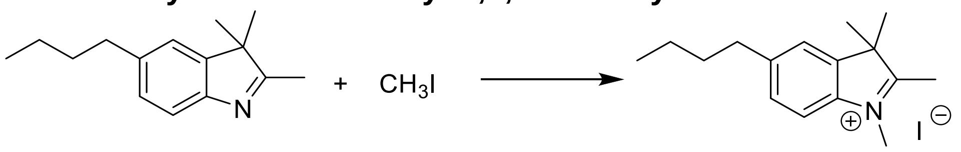

Figure S 2: Schematic representation of the synthesis of compound **2**.

5-*n*-butyl-2,3,3-trimethyl-3*H*-indole (compound 1, 2.0 g, 9.3 mmol) was dissolved in 10 mL acetonitrile, to which methyliodide (1.3 g, 570 µL, 9.3 mmol) was added dropwise in a next step. The solution was heated to reflux for 21 hours under a nitrogen atmosphere. Afterwards, the solvent was removed carefully *via* vacuum distillation to yield compound **2** (3.16 g, 95% yield) as a dark crystalline solid. The resulting compound was used without further purification for the next steps. ^{1}H-NMR ($CDCl_3$, 400 MHz): δ 7.57 (Ar-$\underline{H}$, d, J = 8.25 Hz, 1H), 7.32 (Ar-$\underline{H}$, m, 2H), 4.23 (In-N^+-C$\underline{H}_3$, s, 3H), 3.07 (In-C$\underline{H}_3$, s, 3H), 2.69 (Ar-C$\underline{H}_2$-CH_2-CH_2-CH_3, t, J = 7.82 Hz, 2H), 1.62 (Ar-CH_2-CH_2-C$\underline{H}_2$-CH_3 and Indol-$(CH_3)_2$, m, 8H), 1.35 (Ar-CH_2-CH_2-C$\underline{H}_2$-CH_3, quint., J = 7.39 Hz, 2H), 0.93 (Ar-CH_2-CH_2-CH_2-C$\underline{H}_3$, t, J = 7.34 Hz, 3H). ^{13}C-NMR ($CDCl_3$, 100 MHz): δ 194.6, 145.9, 141.5, 139.9, 129.4, 122.9, 114.9, 54.3, 37.3, 35.7, 33.6, 23.2, 22.3, 17.2, 13.9. HRMS (ESI, m/z): 230.1909 (calc. for $[M-I]^+$ $C_{16}H_{24}N$), found: 230.1922 (Δm/z = + 5.8 ppm).

1.1.3 Synthesis of (4-Hydroxy-5-formylphenyl)-pent-4-yne-1-ol (3)

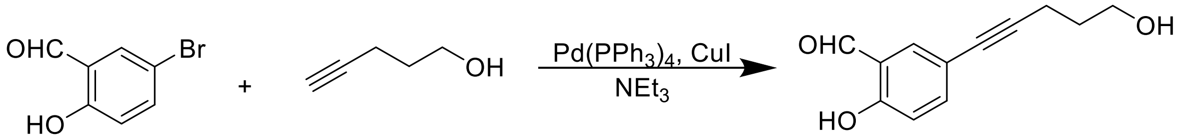

Figure S 3: Schematic representation of the synthesis of compound **3**.

In an oxygen-free atmosphere, 5-Bromosalicylaldehyd (2.40 g, 11.9 mmol), tetrakis(triphenylphosphino) palladium(0) (150 mg, 0.12 mmol) and 55 mg copper(I) iodide (55 mg, 0.29 mmol) were suspended in a mixture of 30 mL THF and triethylamine (3 mL, 4.01 g, 40.1 mmol) in a Schlenk flask. After heating the mixture to 60°C under stirring, pent-4-yne-1-ol (1.00 g, 11.9 mmol) was added to *via* a syringe. The reaction mixture was kept at this

temperature for 16 h. After completion of this procedure, the reaction mixture was cooled to room temperature and the solvent was removed by evaporation. The residue was collected in dichloromethane (DCM) and washed with an aqueous HCl solution (1 N). The CH_2Cl_2 phase was dried over Na_2SO_4, evaporated to dryness and subsequently subjected to column chromatography over SiO_2 using a solvent mixture of ethyl acetate/*n*-hexane/glacial acetic acid = 30:70:1 to yield compound **3** (1.90 g, 78% yield) as an off-white solid. ^{1}H-NMR ($CDCl_3$, 400 MHz): δ 11.04 (Ar-OH, s, br, 1H), 9.85 (-CHO, s, 1H), 7.61 (Ar-H, d, J = 2.09 Hz, 2H), 7.35 (dd, J_1 = 10.76 Hz, J_2 = 6.51 Hz), 6.93 (Ar-H, d, J = 8.62 Hz, 1H), 3.83 (Ar-C≡C-CH_2-CH_2-CH_2OH, t, J = 6.15 Hz, 2H), 2.54 (C≡C-CH_2-CH_2-CH_2OH, t, J = 6.97 Hz, 2H), 1.87 (Ar-C≡C-CH_2-CH_2-CH_2OH, q, J = 6.33 Hz, 2H), 1.74 (C≡C-CH_2-CH_2-CH_2OH, s, 1H). ^{13}C-NMR ($CDCl_3$, 100 MHz): 196.2, 160.9, 139.9, 136.7, 120.4, 117.9, 115.7, 88.9 (C≡C), 79.3 (C≡C), 61.7, 31.4, 15.9. HRMS (EI, positive mode): 204.0786 (calc. for $[M]^+$ $C_{12}H_{12}O_3$), found: 204.0784 (Δm/z = - 1.4 ppm).

1.1.4 5-(5'-*n*-butyl-1',3',3'-trimethylspiro[chromene-2,2'-indolin]-6-yl)pent-4-yn-1-ol (4)

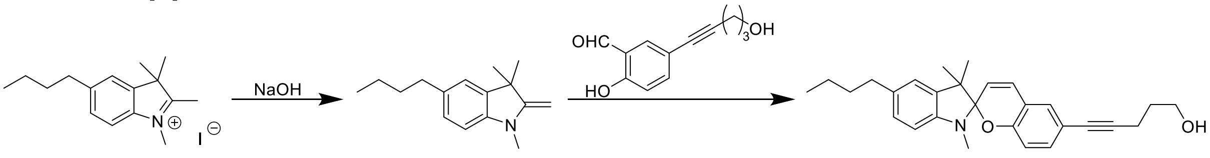

Figure S 4: Schematic representation of the synthesis of compound **4**.

5-butyl-2,3,3-trimethyl-3*H*-indolium iodide (compound **2**, 2.5 g, 7.0 mmol) was added to an aqueous solution of NaOH (3.40 g, 85 mmol dissolved in 20 mL water). The suspension was heated for 15 min to 70°C, whereby a slightly violet liquid separates from the solution as an oil floating up the reaction solution. After cooling, the oil was extracted with diethyl ether from the aqueous phase. The etheric phase was, afterwards, dried over Na_2SO_4 and carefully evaporated to dryness to yield approximately 1.5 g of the intermediate product, which was

directly used for the next synthesis step. Caution: The light-violet oil drastically darkens to dark-violet upon altering. In order to avoid extensive contamination, resulting from the high volatility of this intermediate, flasks for storage need to be sealed with parafilm thoroughly and all glassware in contact with the substance has to be cleaned after utilization. ^{1}H-NMR ($CDCl_3$, 400 MHz): δ 6.97 (2 × Ar-$H^{Indoline}$, m, 2H), 6.49 (Ar-$H^{Indoline}$, d, J = 7.83 Hz, 1H), 3.84 (Indoline=$C\underline{H}_2$, d, J = 3.09 Hz, 2H), 3.05 (Indoline-N-$C\underline{H}_3$, s, 3H), 2.58 (Indoline-$C\underline{H}_2$-CH_2-CH_2-CH_3, t, J = 7.78 Hz, 2H), 1.61 (Indoline-CH_2-CH_2-$C\underline{H}_2$-CH_3, m, 2H), 1.40 (Indoline-$(C\underline{H}_3)_3$ and Indoline-CH_2-$C\underline{H}_2$-CH_2-CH_3, m, 8H), 0.97 (Indoline-CH_2-CH_2-CH_2-$C\underline{H}_3$, t, J = 7.33 Hz, 3H). ^{13}C-NMR ($CDCl_3$, 100 MHz): 163.3, 144.5, 137.6, 133.0, 127.1, 122.1, 104.5, 72.3, 44.2, 35.3, 34.3, 30.0, 28.8, 22.5, 14.0. HRMS (EI, m/z): 229.1830 (calc. for $[M]^+$ $C_{16}H_{23}N$), found: 229.1841 (Δm/z = + 4.8 ppm).

The intermediate was dissolved in 50 mL absolute ethanol along with (4-hydroxy-5-formylphenyl)-pent-4-yne-1-ol (compound **3**, 1.43 g, 7.0 mmol). After degassing the mixture with N_2 for 10 min, it was heated under reflux for 5 h. The dark-red solution was afterwards cooled and evaporated to dryness. The resulting dark-red oil was again dissolved in a small amount of dichloromethane and subjected to column chromatography over SiO_2 using a mixture of ethyl acetate/*n*-heptane/triethylamine = 10:50:1 as an eluent to yield compound **4** (2.34 g, 80% yield) as a yellow oil. Note, that the substance was isolated in its spiropyrane form, which represents the stable form in apolar solvents. Under polar conditions, the substance undergoes ring-opening to yield the corresponding merocyanine form. As the SiO_2 silica gel represents a polar environment, it fosters the transition to the merocyanine (MC) form, if no other apolar solvents are present. Accordingly, the MC state is, *e.g.* formed at a TLC plate after evaporation of the solvents. The spontaneous transition, altering the polarity of the target compound as a result of the absence of apolar solvents, has to be considered during TLC run evaluations. ^{1}H-NMR ($CDCl_3$, 400 MHz): δ 7.16 (Ar-$H^{Chromene}$, d, J = 3.89 Hz, 2H), 7.13 (Ar-$H^{Chromene}$, d, J = 3.89 Hz,

2H), 7.00 (Ar-H^{Indoline}, dd, J_1 = 7.91 Hz, J_2 = 1.62 Hz, 1H), 6.91 (Ar-H^{Indoline}, d, J = 1.50 Hz, 1H), 6.81 (Pyran-H^{Chromene}, d, J = 6.81 Hz, 1H), 6.66 (Ar-H^{Chromene}, d, J = 8.23 Hz, 1H), 6.46 (Ar-H^{Indoline}, d, J = 7.83 Hz, 1H), 5.72 (Pyran-H^{Chromene}, d, J = 10.23 Hz, 1H), 3.84 (Chromene-C≡C-CH_2-CH_2-$C\underline{H}_2$OH, q, J = 5.87 Hz, 2H), 2.72 (Indoline-N-$C\underline{H}_3$, s, 3H), 2.60 (Indoline-$C\underline{H}_2$-CH_2-CH_2-CH_3, t, J = 7.79 Hz, 2H), 2.54 (Chromene-C≡C-$C\underline{H}_2$-CH_2-CH_2OH, t, J = 6.91 Hz, 2H), 1.87 (Chromene-C≡C-CH_2-$C\underline{H}_2$-CH_2OH, t, J = 6.46 Hz, 2H), 1.74 (Chromene-C≡C-CH_2-CH_2-CH_2O$\underline{H}$, t, J =5.46 Hz, 1H), 1.62 (Indoline-CH_2-CH_2-$C\underline{H}_2$-CH_3, m, 2H), 1.41 (Indoline-CH_2-$C\underline{H}_2$-CH_2-CH_3, d, J = 7.51 Hz, 2H), 1.30 (Ar^{Indoline}-$C\underline{H}_3$, s, 3H), 1.18 (Ar^{Indoline}-$C\underline{H}_3$, s, 3H), 0.97 (Indoline-CH_2-CH_2-CH_2-$C\underline{H}_3$, t, J = 7.33 Hz, 3H). ^{13}C-NMR ($CDCl_3$, 100 MHz): 154.3, 146.1, 136.7, 133.9, 133.0, 129.8, 128.8, 127.2, 121.7, 120.2, 118.8, 115.1, 115.0, 106.5, 104.8, 87.4 (C≡C), 80.9 (C≡C), 61.9, 51.9, 46.2, 35.4, 34.2, 31.5, 29.0, 26.0, 22.6, 20.2, 16.0, 14.1. HRMS (EI, m/z): 415.2511 (calc. for $[M]^+$ $C_{28}H_{33}NO_2$), found: 415.2501 (Δm/z = - 2.5 ppm).

1.1.5 5-(5'-butyl-1',3',3'-trimethylspiro[chromene-2,2'-indolin]-6-yl)pent-4-yn-1-ol methyl sulfonic acid ester (5)

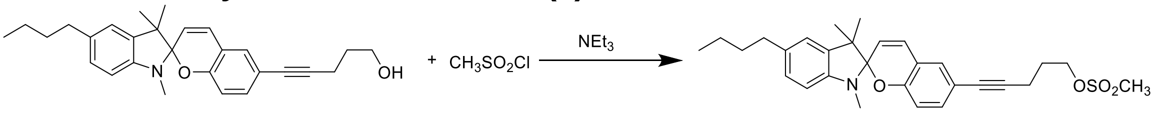

Figure S 5: Schematic representation of the synthesis of compound **5**.

Compound **4** (2.00 g, 4.8 mmol) was dissolved in 150 mL dry DCM. To this solution, 5 mg 4-(*N*,*N'*-dimethylamino)pyridine and triethylamine (1.33 mL, 965 mg, 9.6 mmol, 2 eq.) was added. Under cooling in an ice bath, methanesulfonic chloride (578 mg, 5.1 mmol, 1.05 eq.) was added dropwise. During this addition, the previously yellow solution turned light-red. After removal of the ice bath, the solution was stirred at room temperature for 22 h. During this reaction, further 420 µL triethylamine were added. After the reaction time, the dichloromethane solution was

extracted with an aqueous solution of HCl (0.1 N), whereby the red color of the solution further intensifies. Drying of the DCM phase over Na_2SO_4 and further evaporation to dryness yielded compound **5** (2.247 g, 94.5% yield) as a violet oil, which was used without further purification. ^{1}H-NMR ($CDCl_3$, 400 MHz): δ 7.13 (2 × Ar-$H^{Chromene}$, m, 2H), 7.00 (Ar-$H^{Indoline}$, dd, J_1 = 9.48 Hz, J_2 = 1.63 Hz, 1H), 6.90 (Ar-$H^{Indoline}$, d, J = 1.52 Hz, 1H), 6.81 (Pyran-$H^{Chromene}$, d, J = 10.24 Hz, 1H), 6.66 (Ar-$H^{Chromene}$, d, J = 8.04 Hz, 1H), 6.46 (Ar-$H^{Indoline}$, d, J = 7.84 Hz, 1H), 5.72 (Pyran-$H^{Chromene}$, d, J = 10.24 Hz, 1H), 4.43 (Chromene-C≡C-CH_2-CH_2-$C\underline{H}_2OSO_2CH_3$, t, J = 6.12 Hz, 2H), 3.06 (Chromene-C≡C-CH_2-CH_2-$CH_2OSO_2C\underline{H}_3$, s, 3H), 2.71 (Indoline-N-$C\underline{H}_3$, s, 3H), 2.58 (Indoline-$C\underline{H}_2$-CH_2-CH_2-CH_3 and Chromene-C≡C-$C\underline{H}_2$-CH_2-CH_2 OSO_2CH_3, m, 4H), 2.04 (Chromene-C≡C-CH_2-$C\underline{H}_2$-$CH_2OSO_2CH_3$, t, J = 6.37 Hz, 2H), 1.62 (Indoline-CH_2-CH_2-$C\underline{H}_2$-CH_3, m, 2H), 1.41 (Indoline-CH_2-$C\underline{H}_2$-CH_2-CH_3, m, 2H), 1.29 ($Ar^{Indoline}$-$C\underline{H}_3$, s, 3H), 1.17 ($Ar^{Indoline}$-$C\underline{H}_3$, s, 3H), 0.96 (Indoline-CH_2-CH_2-CH_2-$C\underline{H}_3$, t, J = 7.29 Hz, 3H). ^{13}C-NMR ($CDCl_3$, 100 MHz): 154.5, 146.1, 136.7, 133.9, 133.0, 129.9, 128.7, 127.2, 121.7, 120.3, 118.9, 115.2, 114.7, 106.5, 104.7, 85.6 (C≡C), 81.7 (C≡C), 68.6, 51.9, 37.3, 35.4, 34.2, 29.0, 28.2, 26.0, 22.6, 20.2, 15.7, 14.1. HRMS (EI, m/z): 493.2287 (calc. for $[M]^+$ $C_{29}H_{35}NO_4{}^{[32]}S$), found: 493.2280 (Δm/z = - 1.5 ppm).

1.1.6 5-(5'-butyl-1',3',3'-trimethylspiro[chromene-2,2'-indolin]-6-yl)pent-4-yn-1-bromide (6)

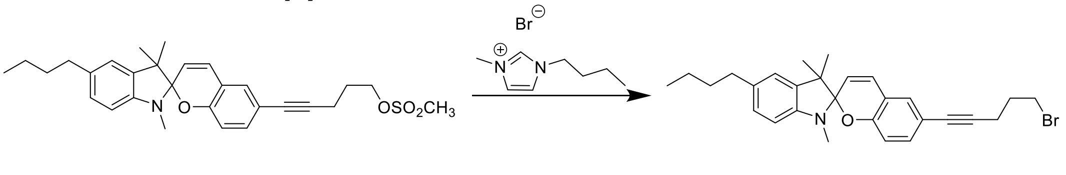

Figure S 6: Schematic representation of the synthesis of compound **6**.

To achieve a bromination, compound **5** (2.0 g, 4.05 mmol) was added along with 1-butyl-3-methylimidazolium bromide (2.0 g, 9.1 mmol) in a tapered pear-shaped flask. The mixture was heated to 60°C, whereupon all components molt together. After 2 hours exposure to these conditions, the dark-red mixture was allowed to cool down. The solidified mixture was collected in dichloromethane and extracted with water. The organic phase was dried over Na_2SO_4,

evaporated to dryness and chromatographied over silica gel eluating with a mixture of ethyl acetate/*n*-hexane = 1:8 to yield compound **6** (1.255 g, 55% yield) as a light-violet oil.

^{1}H-NMR ($CDCl_3$, 400 MHz): δ 7.15 (2 × Ar-H^{Chromene}, m, 2H), 7.02 (Ar-H^{Indoline}, d, dd, J_1 = 7.85 Hz, J_2 = 1.63 Hz, 1H), 6.92 (Ar-H^{Indoline}, d, J = 1.48 Hz, 1H), 6.82 (Pyran-H^{Chromene}, d, J = 10.24 Hz, 1H), 6.68 (Ar-H^{Chromene}, d, J = 8.23 Hz, 1H), 6.47 (Ar-H^{Indoline}, d, J = 7.83 Hz, 1H), 5.73 (Pyran-H^{Chromene}, d, J = 10.23 Hz, 1H), 3.61 (Chromene-C≡C-CH_2-CH_2-C$\underline{H}_2$Br, t, J = 6.51 Hz, 2H), 2.73 (Indoline-N-C$\underline{H}_3$, s, 3H), 2.61 (Indoline-C$\underline{H}_2$-CH_2-CH_2-CH_3 and Chromene-C≡C-C$\underline{H}_2$-CH_2-CH_2Br, m, 4H), 2.15 (Chromene-C≡C-CH_2-C$\underline{H}_2$-CH_2Br, quint., J = 13.22 Hz, 2H), 1.62 (Indoline-CH_2-CH_2-C$\underline{H}_2$-CH_3, m, 2H), 1.42 (Indoline-CH_2-C$\underline{H}_2$-CH_2-CH_3, q, J = 7.53 Hz, 2H), 1.31 (Ar^{Indoline}-C$\underline{H}_3$, s, 3H), 1.19 (Ar^{Indoline}-C$\underline{H}_3$, s, 3H), 0.98 (Indoline-CH_2-CH_2-CH_2-C$\underline{H}_3$, t, J = 14.67 Hz, 3H). ^{13}C-NMR ($CDCl_3$, 100 MHz): 154.4, 146.1, 136.7, 133.9, 133.1, 129.9, 128.7, 121.7, 120.3, 118.8, 115.2, 114.9, 106.5, 104.8, 85.9 (C≡C), 81.3 (C≡C), 51.8, 43.8, 35.4, 34.2, 32.5, 31.7, 29.0, 26.0, 22.6, 20.2, 18.2, 14.2. HRMS (EI, m/z): 477.1667 (calc. for $[M]^+$ $C_{28}H_{32}NO^{[79]}Br$), found: 477.1645 (Δm/z = - 4.6 ppm).

1.1.7 5-(5'-butyl-1',3',3'-trimethylspiro[chromene-2,2'-indolin]-6-yl)pent-4-yn-1-(trimethyl-ammonium) bromide (7)

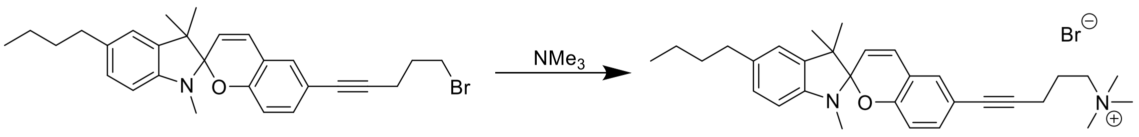

Figure S 7: Schematic representation of the synthesis of compound **7**.

Compound 7 (1.1 g, 2.3 mmol) was dissolved 5 mL of a solution of trimethylamine $N(CH_3)_3$ in ethanol (31-35 %). The greenish solution was heated to 50°C for 4 days. After completion of the reaction, the reaction mixture was diluted with ~ 200 mL of MilliQ water and filtered through a syringe filter (Whatman®, Nylon, 0.45 µm). Freeze-drying of the solution yielded a violet solid,

which was collected in a small amount of ethyl acetate (~ 5 mL) and precipitated in 45 mL *n*-hexane to yield the target compound **7** (1.093 g, 88.4 %) as a yellowish powder. ^{1}H-NMR ($CDCl_3$, 400 MHz): δ 7.17 (Ar-$H^{Chromene}$, d, J = 1.98 Hz, 1H), 7.12 (Ar-$H^{Chromene}$, dd, J_1 = 8.63 Hz, J_2 = 2.04 Hz, 1H), 6.98 (Ar-$H^{Indoline}$, dd, J_1 = 7.92 Hz, J_2 = 1.67 Hz, 1H), 6.89 (Ar-$H^{Indoline}$, d, J = 1.52 Hz, 1H), 6.83 (Pyran-$H^{Chromene}$, d, J = 10.27 Hz, 1H), 6.63 (Ar-$H^{Chromene}$, d, J = 8.39 Hz, 1H), 6.43 (Ar-$H^{Indoline}$, d, J = 7.84 Hz, 1H), 5.70 (Pyran-$H^{Chromene}$, d, J = 10.23 Hz, 1H), 3.73 (Chromene-C≡C-CH_2-CH_2-$C\underline{H}_2$-$N^+(CH_3)_3$, m, 2H), 3.49 (Chromene-C≡C-CH_2-CH_2-CH_2-$N^+(C\underline{H}_3)_3$, s, 1H), 2.69 (Indoline-N-$C\underline{H}_3$, s, 3H), 2.59 (Indoline-$C\underline{H}_2$-CH_2-CH_2-CH_3 and Chromene-C≡C-$C\underline{H}_2$-CH_2-$CH_2N^+(CH_3)_3$, m, 4H), 2.04 (Chromene-C≡C-CH_2-$C\underline{H}_2$-$CH_2N^+(CH_3)_3$, m, 2H), 1.60 (Indoline-CH_2-CH_2-$C\underline{H}_2$-CH_3, d, m, 2H), 1.38 (Indoline-CH_2-$C\underline{H}_2$-CH_2-CH_3, q, J = 7.53 Hz, 2H), 1.27 ($Ar^{Indoline}$-$C\underline{H}_3$, s, 3H), 1.15 ($Ar^{Indoline}$-$C\underline{H}_3$, s, 3H), 0.95 (Indoline-CH_2-CH_2-CH_2-$C\underline{H}_3$, t, J = 14.65 Hz, 3H). ^{13}C-NMR ($CDCl_3$, 100 MHz): 154.6, 146.1, 136.5, 133.9, 133.1, 130.1, 128.7, 127.2, 121.7, 120.4, 118.8, 115.2, 114.2, 106.5, 104.9, 84.9 (C≡C), 82.4 (C≡C), 66.2, 53.7, 51.9, 35.4, 34.2, 29.0, 25.9, 22.6, 20.1, 17.1, 14.0. HRMS (ESI, m/z): 457.3219 (calc. for $[M\text{-}Br]^+$ $C_{31}H_{41}N_2O$), found: 457.3183 (Δm/z = - 7.8 ppm).

1.2 Photophysical characterization of the system

Time resolved UV/Vis measurements were conducted with a commercial Cary 5000 UV/Vis-NIR spectrophotometer instrument (Agilent Technologies, USA). For this purpose, a quartz cuvette with a rectangular shape (commercial cuvette from Helma Analytics, transparent in all directions), with 1 cm light patch is filled with 1.8 mL of the sample solution. A light source with the respective excitation wavelength is placed near the sample holder with an illumination light path oriented perpendicularly to the measurement channel. The LED lamp (Thorlabs), thereby, irradiates the total volume of the sample holder. The data was recorded for the set of different

intensities for all fixed wavelength, *i.e.* λ = 365 nm, λ = 455 nm, λ = 490 nm, and λ = 530 nm. The light intensities are directly measured at the sample position prior to each measurement using a commercial S170C power meter (Thorlabs).
Fluorescence emission spectra were recorded with a Tecan Plate Reader Infinite200Pro (Tecan, Switzerland). For this reason, excitation wavelengths with 435 nm (Figure 2(a)) and 550 nm (Figure 2(b)), respectively, were chosen. For representation, the spectra shown in Figure 2 (a) and (b) in the main part of the manuscript were smoothed.

1.3 Potentiometric titration of compound 7

The potentiometric titration was conducted in a 80 mL glass beaker as a titration cell with a general-purpose pH probe connected to a Mettler Toledo pH-meter. Prior to each experiment, the pH-meter was thoroughly washed with deionised water and calibrated with standard buffers (pH 4.0, pH 7.0 and pH 10.0). All solutions were prepared with deionised water.
The titrate solution (50 mL), prepared by dissolving the surfactant (0.2 mM) in trifluoroacetic acid (TFA, ≥ 99.9% Carl Roth, 1 mM), previously heated up (50°C) or illuminated with blue light (455 nm, 1A THORLABS® lamp) for 30 min, was transferred to the titration cell. TFA (1 mM) was used as blank titrate solution. All titrations were carried out at ambient temperature (25°C) and under gentle mixing with a magnetic stirrer. For the surfactant previously irradiated, the titration was specifically performed under constant illumination with blue light.
The titrant solution, freshly prepared NaOH (1 M), was gradually added with Eppendorf micro pipettes (from 2.5 to 90 µL). Sufficient time (about 1 min) was necessary to reach a stable pH reading before the next addition of base. The recorded pH values were plotted against the

cumulative volumes of titrant. The titration curve and its first derivative were used to determine the equivalence points as well as the pK_a.

1.4 Determination of the critical micelle concentration

To determine the respective critical micelle concentrations (CMCs) of the surfactant, the concentration-dependent surface tension of an aqueous solution was determined. For this purpose, a concentration series of compound 7 was prepared (5 µM to 10 mM for neutral and alkaline conditions and extension of this range to 150 mM for acidic conditions). Surface tension measurements were conducted as pendant-drop measurements using a contact angle measurement system (dataphysics OCA-15, dataphysics, Germany), and the data were evaluated with the dataphysics SCA202 software. The measurements were performed at a pH of approx. 10, buffering with a NaOAc/NaOH (c ~ 10^{-4} M) aqueous solution, in MilliQ water, and at a pH of 4, where the surfactant was protonated by adding one equivalent of TFA and dissolved in a TFA solution (c = 10^{-4} M). These solutions were measured either in their thermodynamically relaxed state (water bath at 60°C for 15 minutes, afterward cooling to room temperature) and under photo-illumination using the aforementioned LED lamp with 455 nm excitation wavelength for 10-20 minutes prior to the measurements and during the measurement, whereby the pending drop was irradiated for further 2 min.

1.5 Computational details

Density functional theory (DFT) calculations were performed using the Gaussian 16 program.[1] The B3LYP functional[2,3] in combination with the def2-TZVP basis set[4] was used. The D3 empirical dispersion correction with Becke–Johnson (BJ) damping was applied.[5] Solvent

(water) effects were accounted for by means of the polarizable continuum model (PCM).[6,7] Surfactant geometries were optimized in the electronic ground state. Normal mode analysis was performed to assess the nature of the optimized geometries, namely zero imaginary frequencies were present for minima and one imaginary frequency for transition states (TS). For the latter, the normal mode corresponding to the imaginary frequency was visually inspected to confirm that this mode corresponds to proper atomic movements (ring-opening or isomerization). Furthermore, intrinsic reaction coordinate (IRC) calculations[8,9] have been conducted starting from the found TS structures to verify the nature of the reactions and locate reactants and products, which were then fully reoptimized. Preliminary TS searches and IRC calculations were performed with a smaller 6-31G* basis set.[10] The B3LYP+D3(BJ)/6-31G*/PCM (water) structures were reoptimized at the B3LYP+D3(BJ)/def2-TZVP/PCM (water) level. Optimizations in gas phase at the B3LYP+D3(BJ)/def2-TZVP level were performed for SP and MC forms for comparison with the calculations using PCM (water). In all calculations, the spin-restricted formalism was used. Alongside the cationic forms of the surfactant, we have also considered protonated, dicationic forms of MC (MCH^+) and SP (SPH^+), which were again optimized at the B3LYP+D3(BJ)/def2-TZVP/PCM (water) level. Thermochemical analyses were performed at a temperature of 298.15 K.

1.6 Emulsification experiments

1.6.1 Photophysical characterization of the emulsions

Oil-in-water emulsions were prepared from toluene (ChromoSolv, >99.5%) and aqueous solutions of the surfactant **7**. For this purpose, first, two stock solutions of compound **7** were prepared, exhibiting a concentration of 40 µg mL^{-1} in (i) MilliQ water (adjusted to a pH value of 7 to 8 with NaOH) and (ii) 10^{-4} M HCl solution, to which further two equivalents of HCl per

surfactant molecule were added. Diluting both stock solutions with MilliQ water (i) and 10^{-4} M aqueous HCl solution (ii), yielded the desired aqueous surfactant solutions with a final concentration of 0.02 µg mL^{-1}. The pH value of the acidic sample was checked with a pH ribbon (Carl Roth).

Furthermore, cetyltrimethylammonium bromide (CTAB, Roth, >99%) was dissolved in both media, yielding concentrations of 26 µg mL^{-1} surfactant. For emulsification, 4.75 mL of the said surfactant solution (compound **7** acidic and neutral, CTAB neutral) was added along with 0.25 mL toluene into a glass vial (20 mL nominal volume). Under vigorous stirring (550 rpm) and ice cooling, the system was subjected to ultrasonication using a SONOPlus HD 3200 sonotrode (Bandelin, Berlin) with a frontal surface possessing a diameter of 3 mm for 60 s with an overall energy of 1.671 kJ. The UV/Vis absorption spectra were recorded with a commercial Cary 5000 UV/Vis-NIR spectrophotometer instrument (Agilent Technologies, USA), using a quartz cuvette with 1 mm light path. The baseline spectrum was thereby measured against the sample with the CTAB-stabilized emulsion. Light microscopy was performed with a Leica DMI-8 fluorescence microscope using a HC PL FLUOTAR 40×/0.80 dry objective. For this purpose, a drop of the emulsion was deposited on a cover slip (Carl Roth, high precision #1) and covered with a second coverslip. Data evaluation was conducted using the program ImageJ 1.52n (available from https://imagej.nih.gov/ij/download.html).

1.6.2 Photo-manipulation of the emulsions

To demonstrate photo-manipulation of the surfactant, emulsions were prepared using a mixture of CTAB and compound **7**. For this purpose, 3.75 mL of a CTAB solution (26 µg mL^{-1}) was mixed with 1.00 mL of a solution of compound **7** under both, acidic and neutral, pH conditions. The resulting solutions were irradiated with blue light using the LED lamp (λ = 455 nm) for > 10 min.

Under ice-cooling, the solution was subjected to ultrasonication as previously described along with 0.25 mL toluene.

To study the photo-manipulation, the emulsion was image via light microscopy. For this purpose, a drop of the emulsion deposited on a cover slip and covered with a second coverslip was measured with an inverted microscope Olympus IX73 equipped with a light source of various wavelengths. Each of the samples, the neutral one as well as the acidic one, was irradiated for at least 10 minutes with an LED light source. The data are recorded for the set of different intensities for a fixed wavelength, *i.e.* using the LED lamp from Thorlabs as UV (λ = 365 nm, M365LP1) and blue (λ = 455 nm) light. LED lamps are collimated in such a way, that the sample is irradiated globally. Time-resolved images were recorded with a CCD camera (Hamamatsu ORCA-Flash4.0 LT (C11440)) at the rate of 2 frame per second (1fps). Red LED (λ =625nm, M625L1-C1, Thorlabs GmbH) is used for imaging as it does not affect the photo-isomerization of the photosystem. The entire setup is kept in the dark to prevent any possible light exposure from the environment. Images and movies were processed with ImageJ.

1.6.3 Reaction control using emulsion droplets as microreactors

To study the kinetics of the model reaction, emulsions were prepared using CTAB as a stabilizer. Two samples were prepared, (a) containing the reaction mixture of *p*-toluenesulfonyl chloride (Sigma Aldrich, >98%) and 4-(*n*-butyl)aniline (Sigma Aldrich, 97%) and (b) only *p*-toluenesulfonyl chloride.

Preparation of (a):

4.75 mL of the CTAB solution (neutral) were pipetted in a 20 mL-glass vial equipped with a magnetic stirrer. To this solution, 250 µL of a stock solution of sodium acetate (NaOAc 20.0 mg mL^{-1}, Fisher) was added. A solution consisting of 4-(*n*-butyl)aniline (7.8 mg mL^{-1}, 1 eq.) and *n*-dodecane (8.9 mg mL^{-1}, Fluka, 98%, used as an internal standard) in toluene as well as

another solution with *p*-toluenesulfonyl chloride (50 mg mL^{-1}, 5 eq.) in toluene was prepared. Under ice cooling, 0.25 mL of each of the toluene solutions were pipetted to the aqueous phase. The heterogenous mixtures were emulsified using the protocol as previously described.

Preparation of (b):

Emulsion (b) was prepared in the same manner as emulsion (a). However, a mixture of 0.25 mL of a diluted solution of *p*-toluenesulfonyl chloride (10 mg mL^{-1}, 1 eq.) and 0.25 mL of toluene were mixed with 4.75 mL of the CTAB solution, to which 250 µL of NaOAc (4.0 mg mL^{-1}) were added.

The reaction progress in both emulsions (a) and (b) was monitored *via* pH using a SenTix HW electrode (WTW) and with gas chromatography coupled with mass spectrometry (GC/MS). The latter was performed with a Hewlett Packard HP 6890 series equipped with an Agilent 19091S-43S column with 5% phenylmethylsiloxane, 30 m×250 µm×0.25 µm with 0.8 mL min^{-1} Helium stream, using a temperature of 70°C for 2 min followed by a temperature ramp with 20°C min^{-1} for ten minutes and a subsequent constant temperature of 230°C. The gas chromatograph was coupled with a 5973Network mass selective detector (Agilent Technologies). For gas chromatography, after different time points of the reaction, 2 mL of the emulsion was sampled, filled in a glass vial, and a mixture of 1.5 mL dichloromethane and 1 mL brine was added. After vigorous shaking, the emulsion became a stable two-phase system, from which the DCM phase was subjected to GC/MS analysis.

1.6.4 Targeted destabilization of the emulsion droplets used as microreactors

For this purpose, the two emulsions (a) and (b) were prepared in analogy to the previous section. Here, the aqueous solutions consist of 3.75 mL of CTAB solution (26 µg mL^{-1}) and 1.00 mL of a solution of compound **7** (40 µg mL^{-1}), and solutions of *p*-toluenesulfonyl chloride (50 mg mL^{-1},

5 eq., for emulsion (a) and 10 mg mL^{-1} for emulsion (b)) and 4-(*n*-butyl)aniline (7.9 mg mL^{-1}, 1 eq.; no amount of *n*-dodecane was added) for (b).

Compound 7 was exposed to irradiation with blue light prior to the emulsification process. Ultrasonication was performed under ice cooling as previously explained. Emulsion (a) was subjected to demulsification with UV light after 2 h and (b) after 2 and 8 h using the conditions as described in 1.6.2.

2. Supplementary spectra characterizing the target compound and its intermediates

In this section, a supplementary NMR and ESI-MS spectra are provided for the characterization of the compounds **1 – 7**.

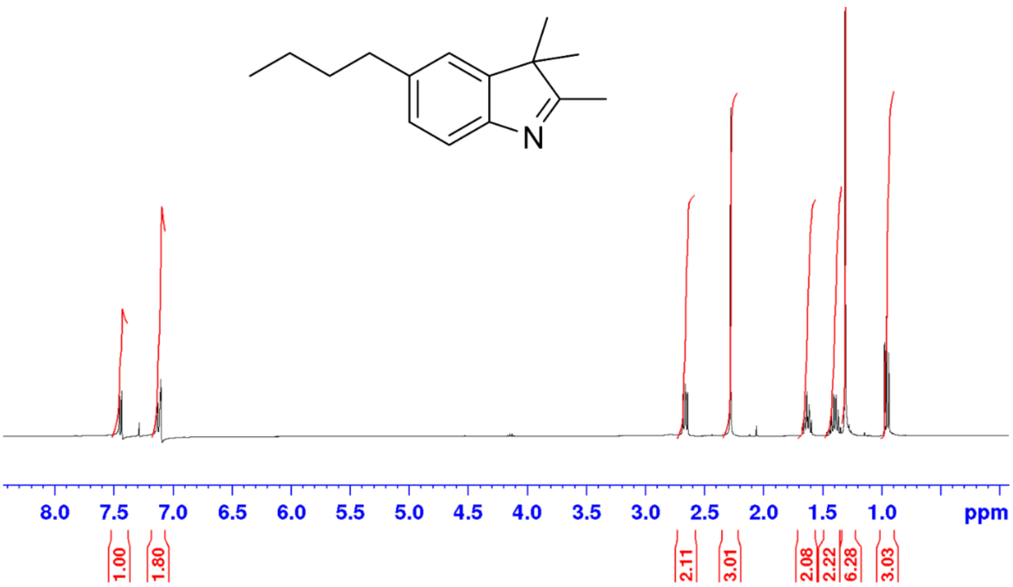

Figure S 8: ^{1}H-NMR spectrum of compound 1 (400 MHz, $CDCl_3$).

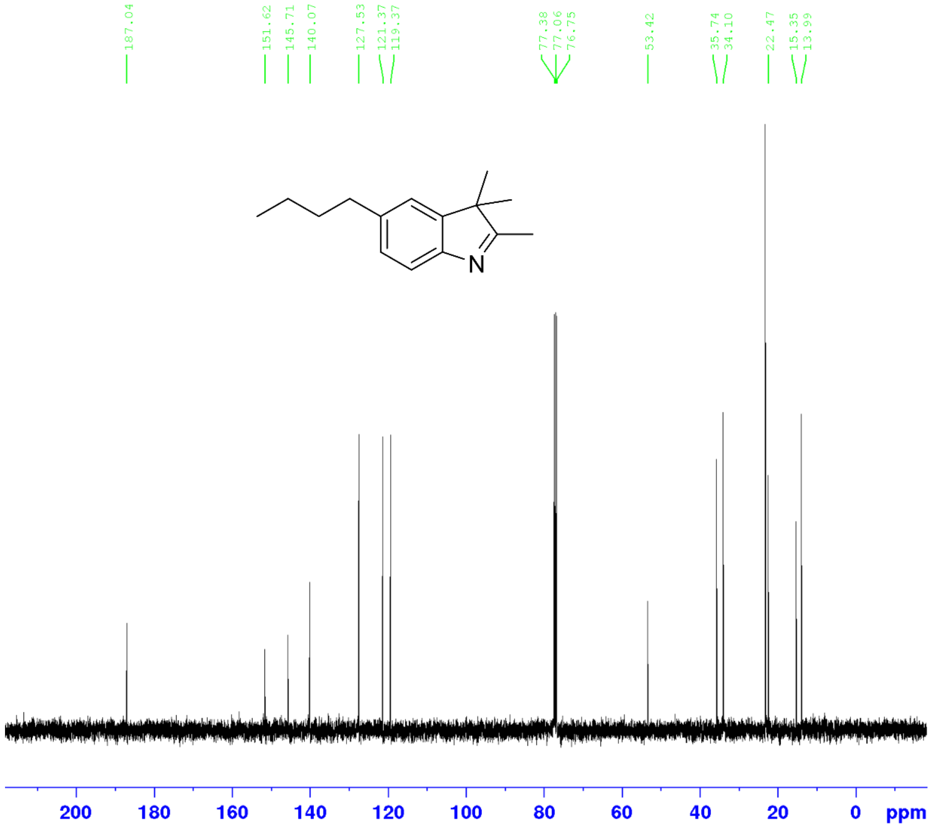

Figure S 9: ^{13}C-NMR spectrum of compound 1 (100 MHz, $CDCl_3$).

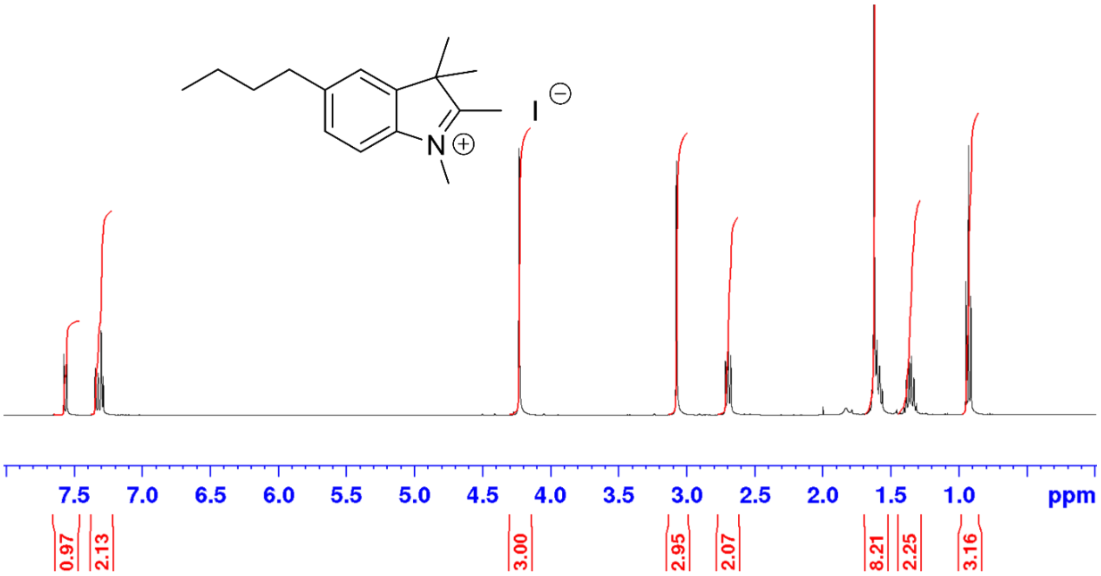

Figure S 10: ^{1}H-NMR spectrum of compound 2 (400 MHz, $CDCl_3$).

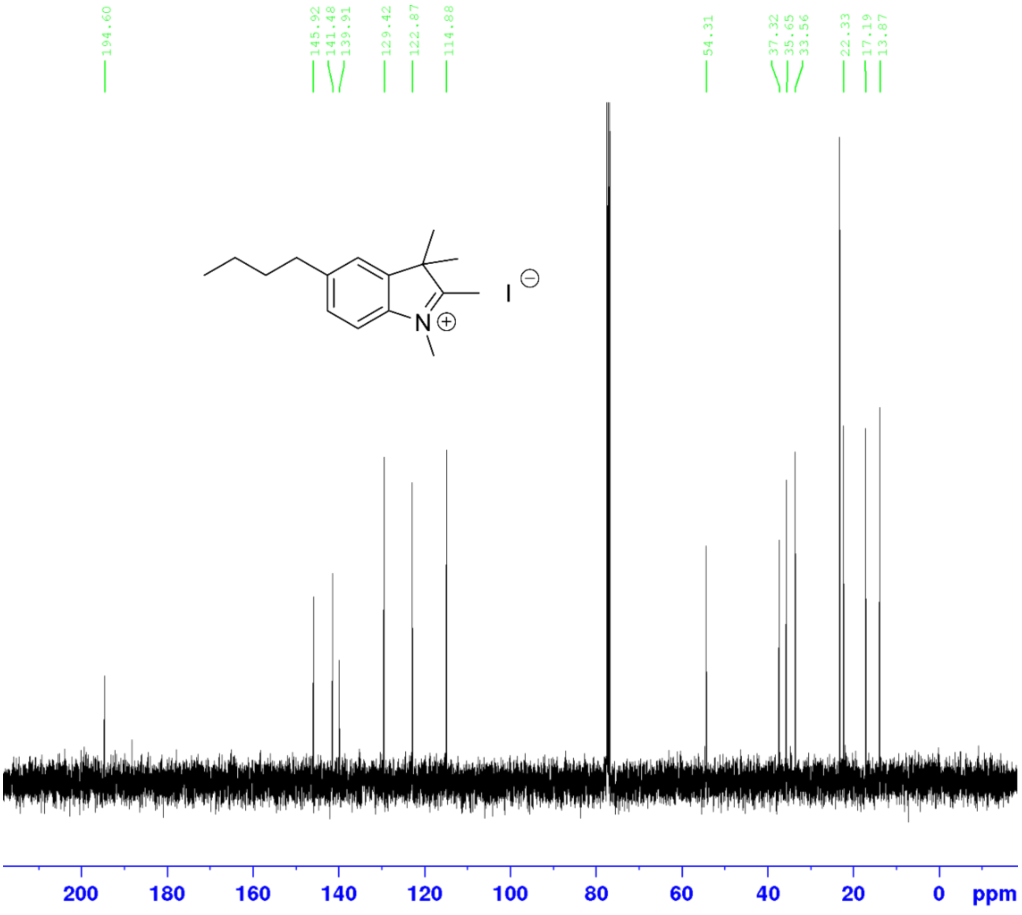

Figure S 11: ^{13}C-NMR spectrum of compound 2 (100 MHz, $CDCl_3$).

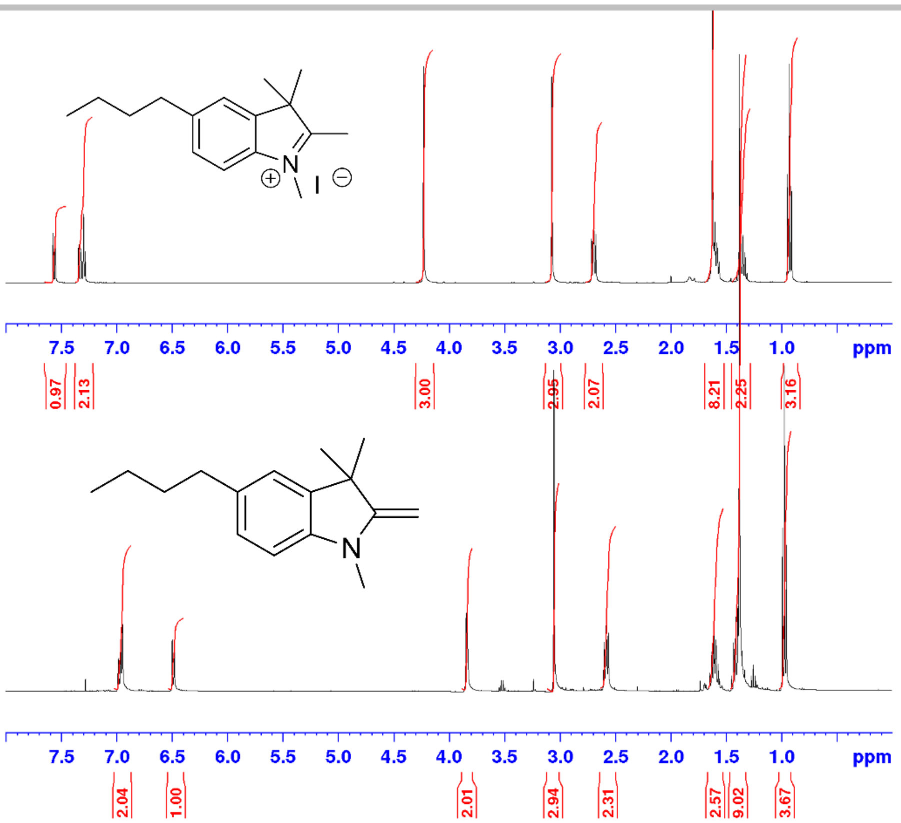

Figure S 12: ^{1}H-NMR spectra monitoring the deprotonation reaction of compound 2 (400 MHz, $CDCl_3$). The intermediate was not worked up.

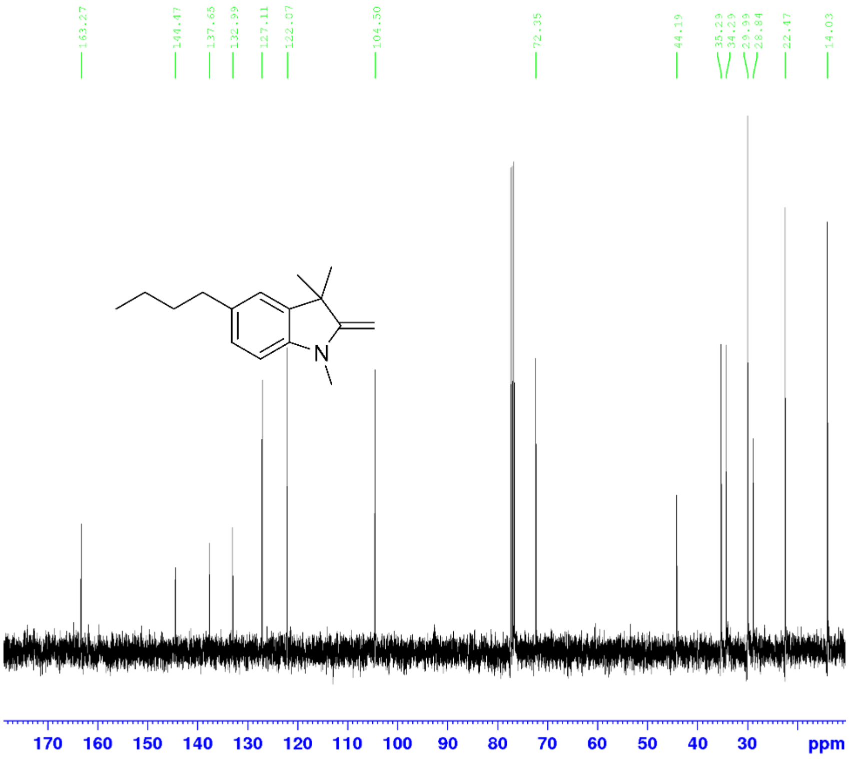

Figure S 13: ^{13}C-NMR spectra of the deprotonation product of compound 2 (400 MHz, $CDCl_3$). The intermediate was not worked up.

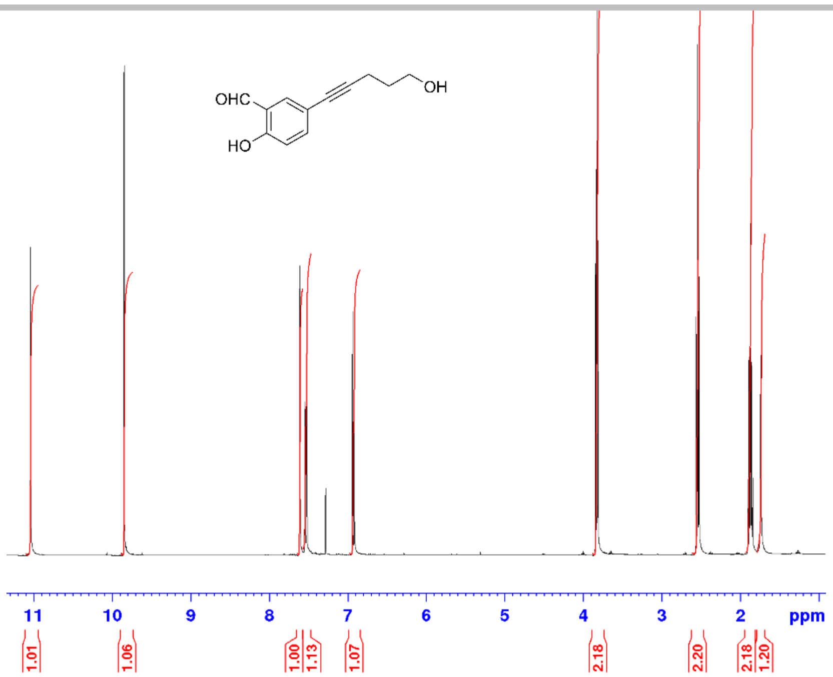

Figure S 14: ^{1}H-NMR spectrum of compound *3* (400 MHz, $CDCl_3$).

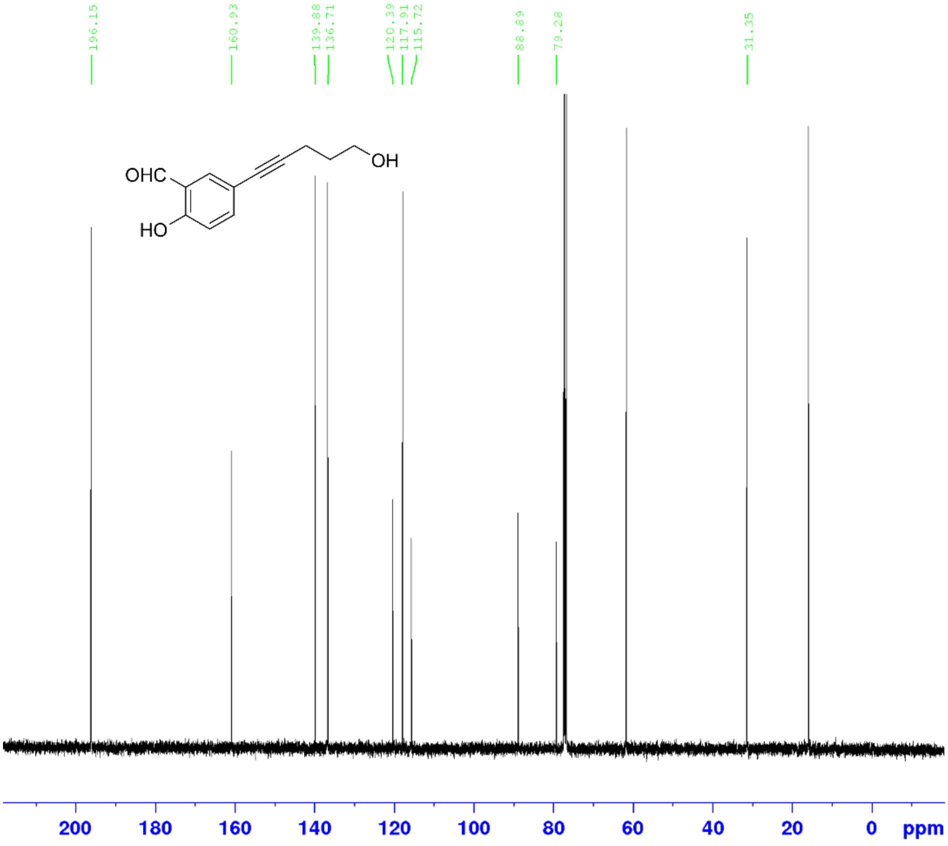

Figure S 15: ^{13}C-NMR spectrum of compound 3 (100 MHz, $CDCl_3$).

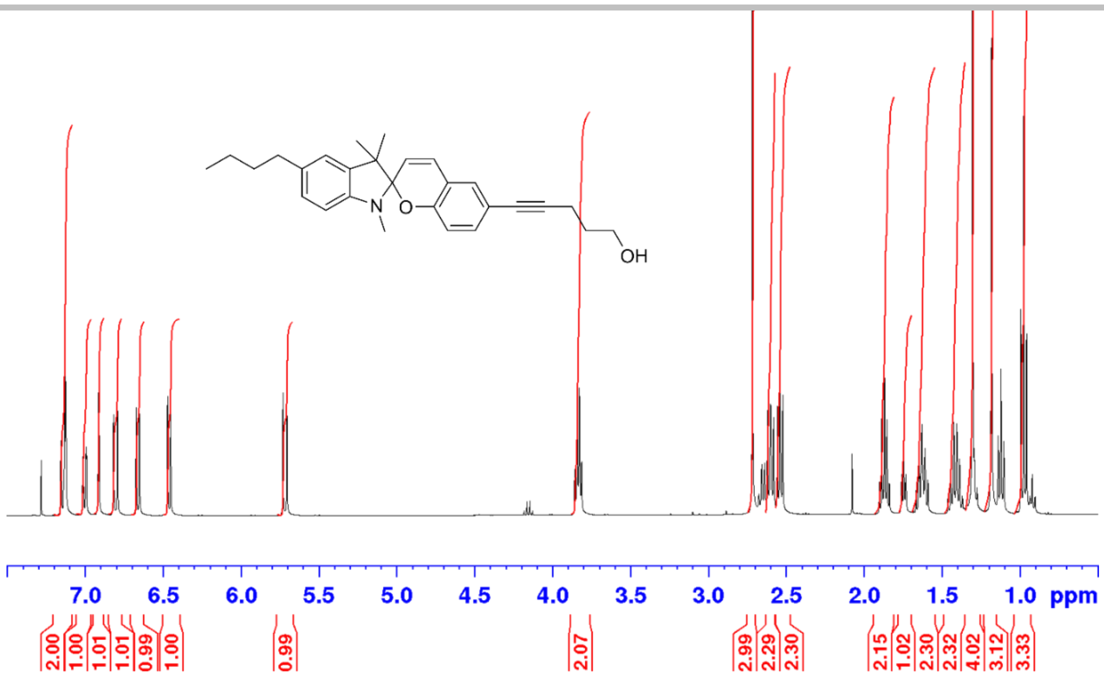

Figure S 16: ^{1}H-NMR spectrum of compound 4 (400 MHz, $CDCl_3$).

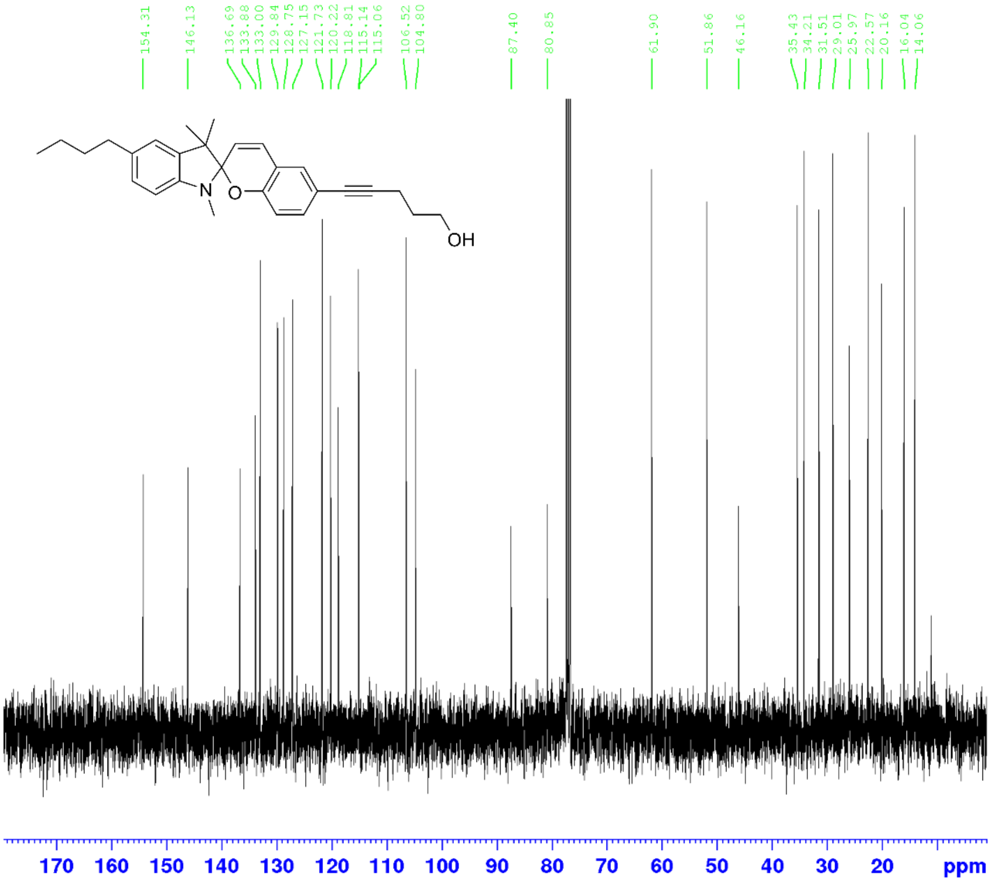

Figure S 17: ^{13}C-NMR spectrum of compound 4 (100 MHz, $CDCl_3$).

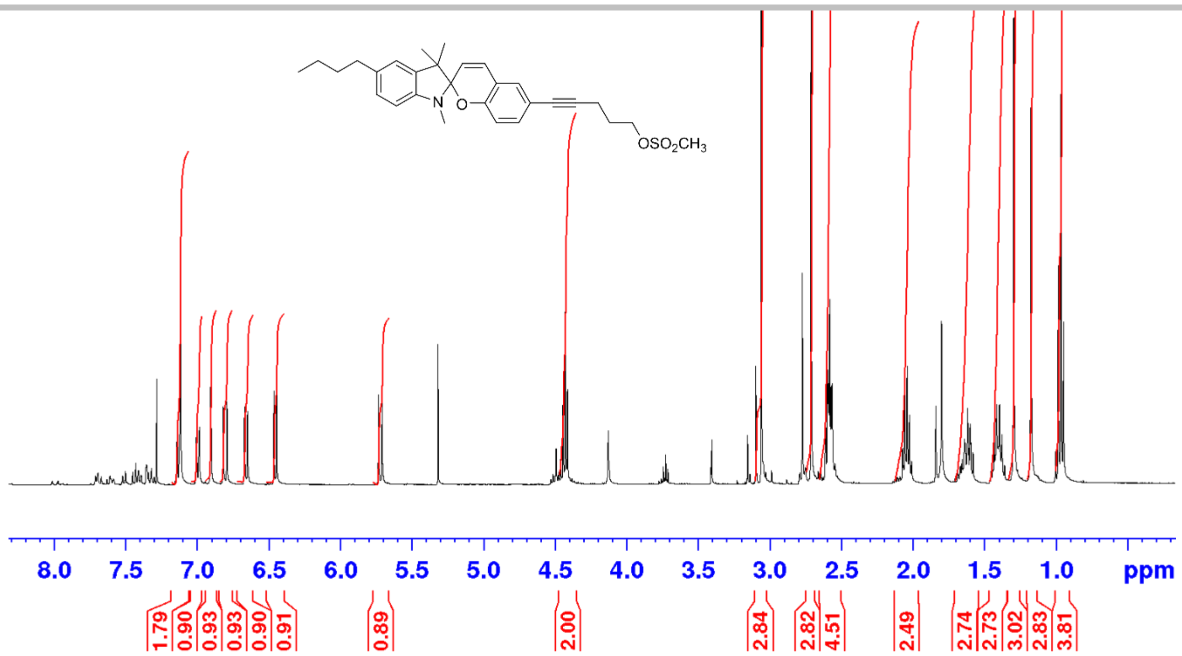

Figure S 18: ^{1}H-NMR spectrum of compound 5 (400 MHz, $CDCl_3$).

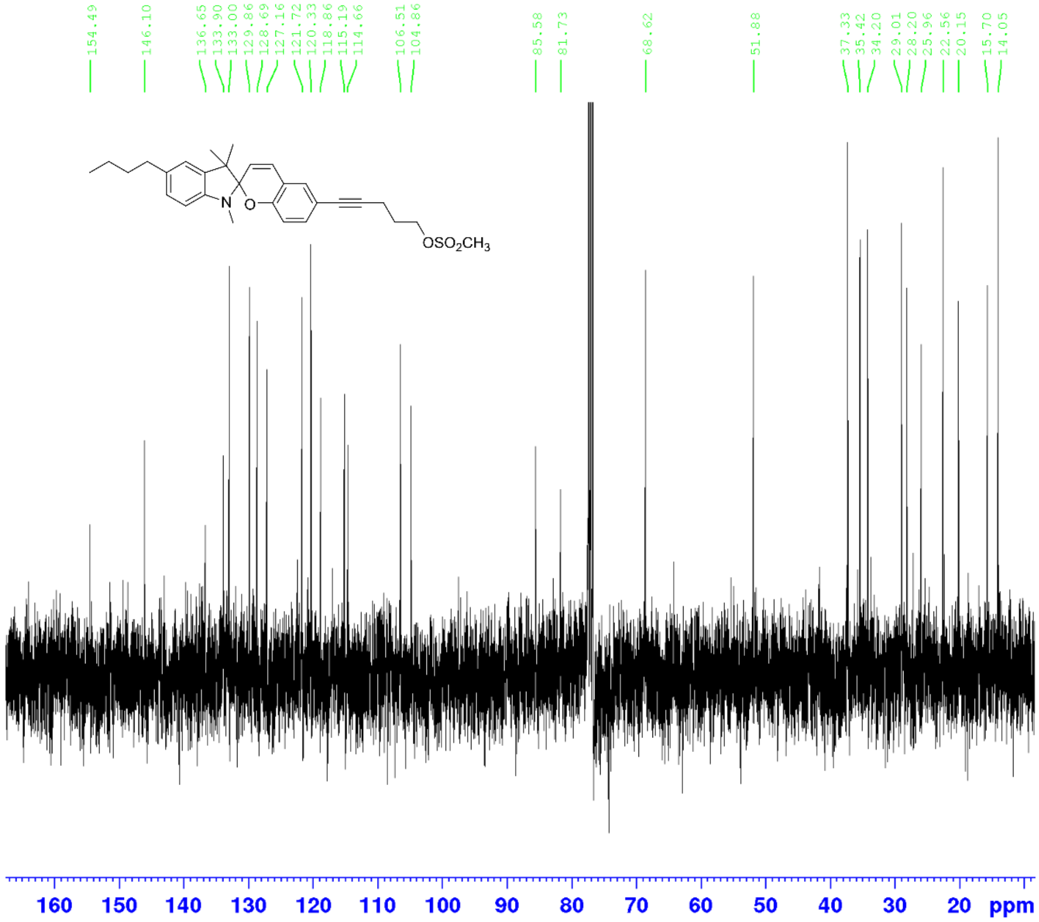

Figure S 19: ^{13}C-NMR spectrum of compound 5 (100 MHz, $CDCl_3$).

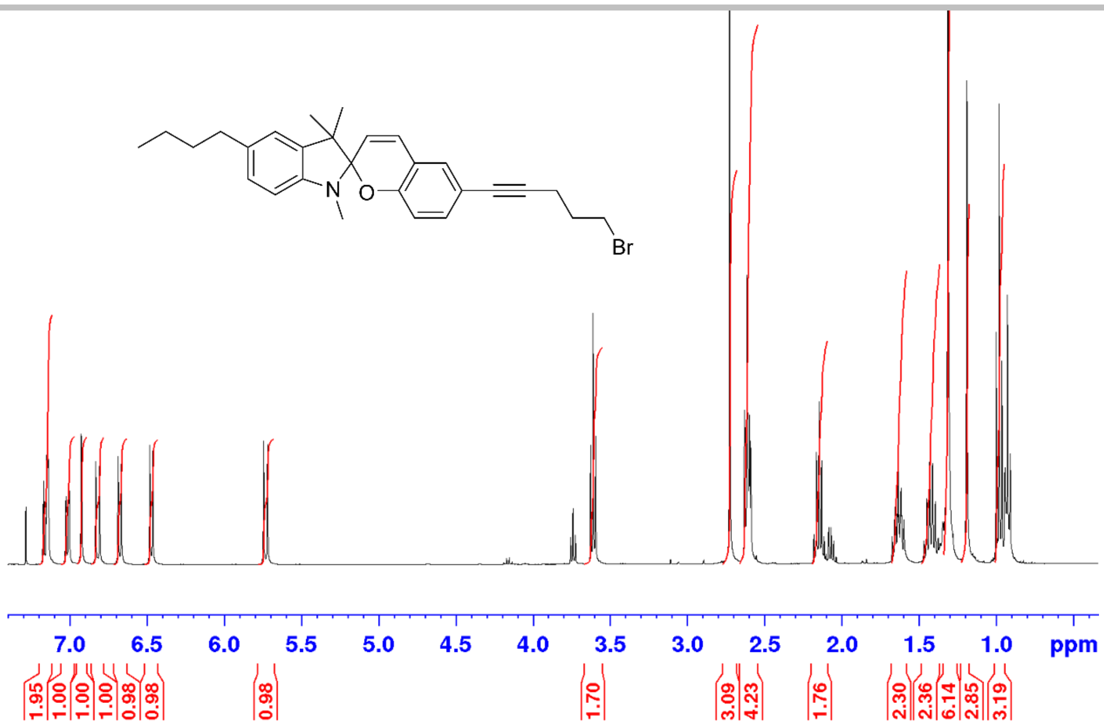

Figure S 20: ^{1}H-NMR spectrum of compound 6 (400 MHz, $CDCl_3$).

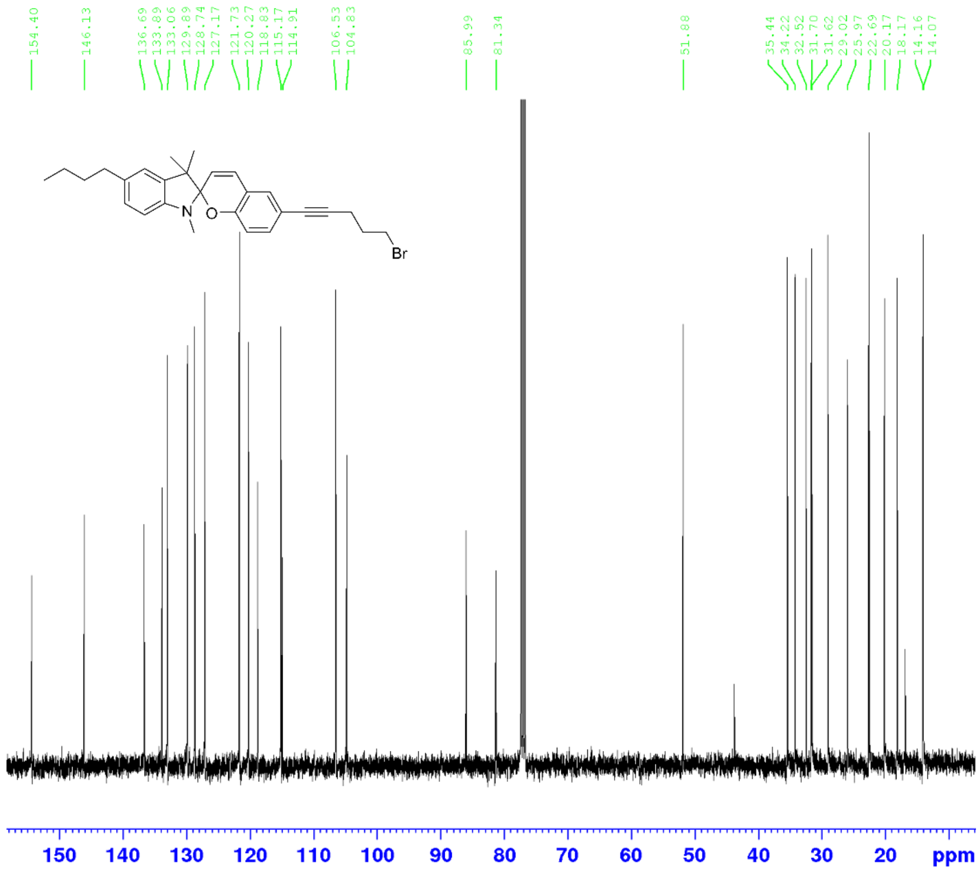

Figure S 21: ^{13}C-NMR spectrum of compound 6(100 MHz, $CDCl_3$).

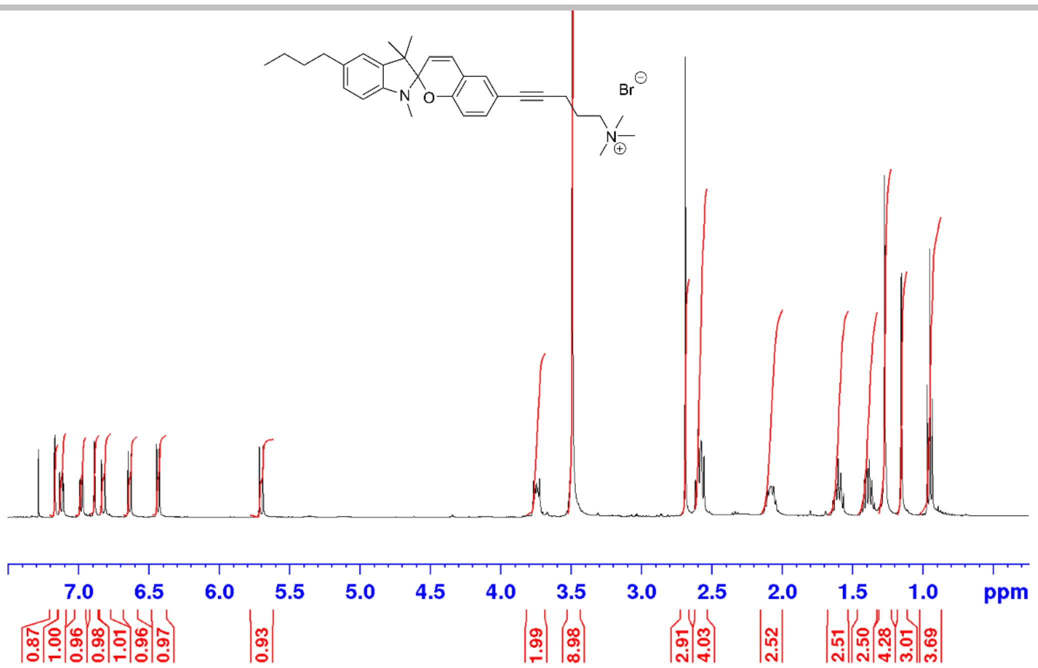

Figure S 22: ^{1}H-NMR spectrum of compound 7 (400 MHz, $CDCl_3$).

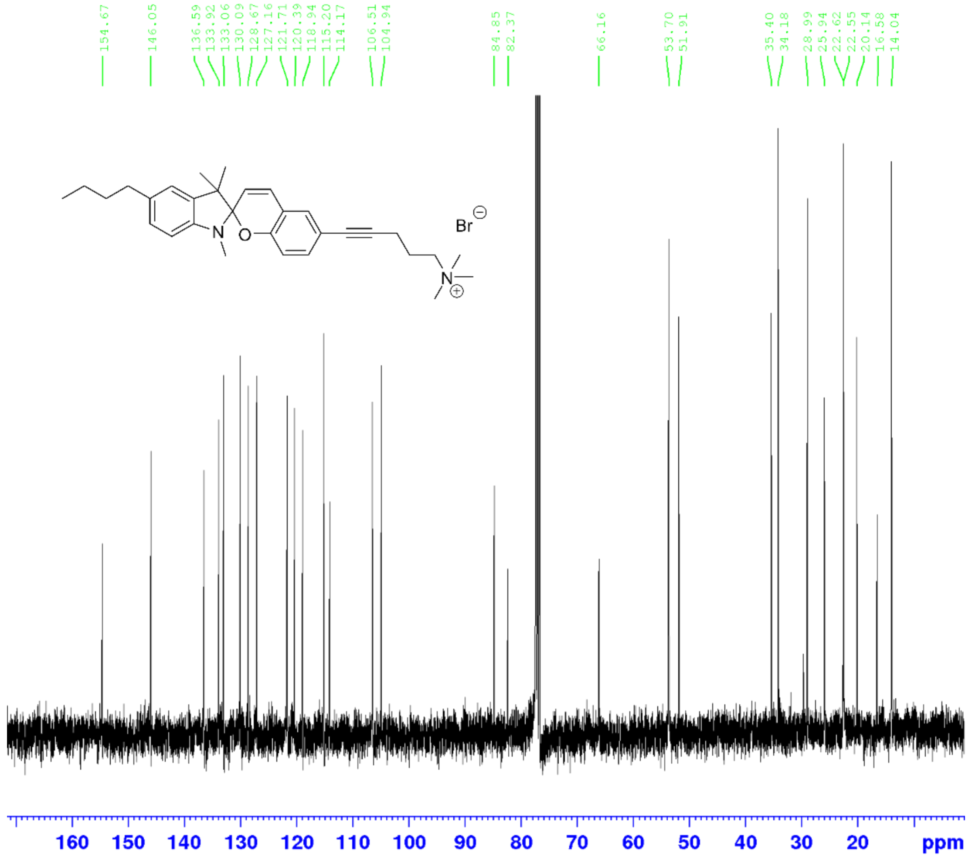

Figure S 23: ^{13}C-NMR spectrum of compound 7 (100 MHz, $CDCl_3$).

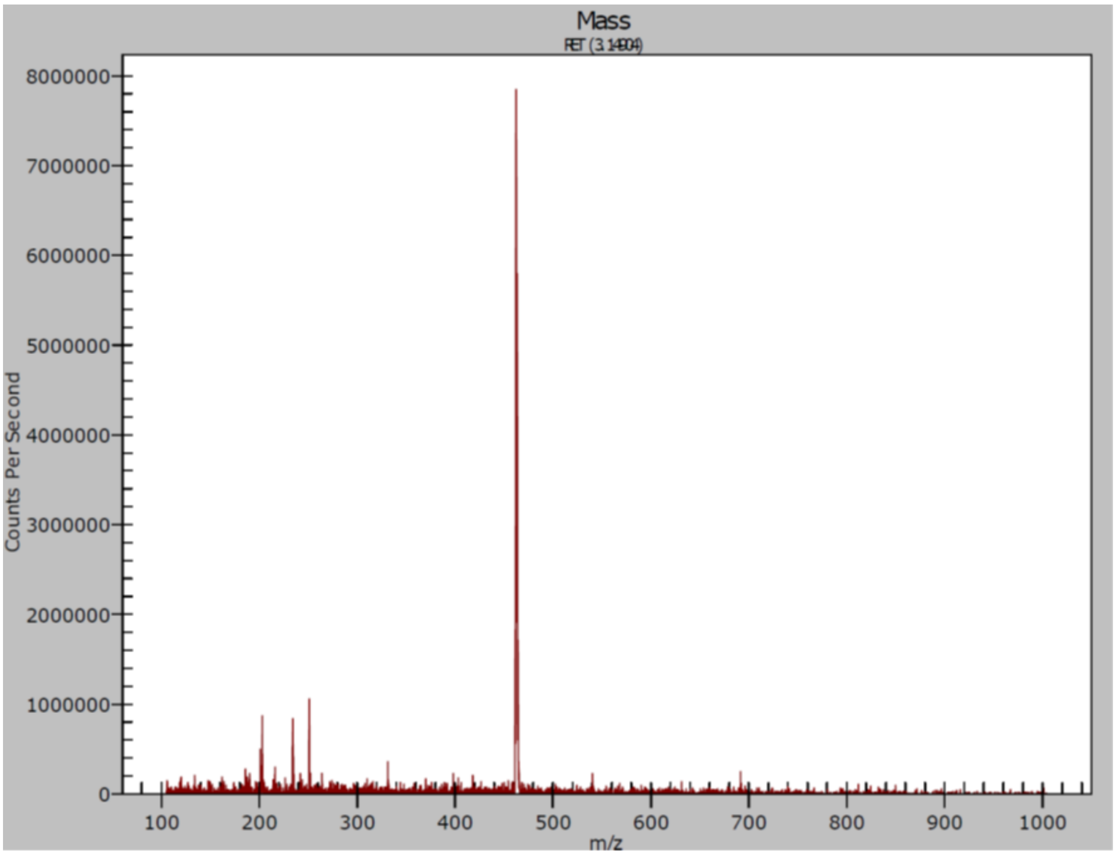

Figure S 24: ESI-MS spectrum of the final product compound **7**, recorded in positive mode. The spectrum suggests a high purity of the final compound. The cationic surfactant possesses $[M]^+$ with 457 g mol^{-1}.

3. Photophysical investigations

This section provides an overview about supplementary details of the photophysical characterization of compound **7**. These were performed using LED lamps, whose emission characteristics are displayed in Figure S *25*.

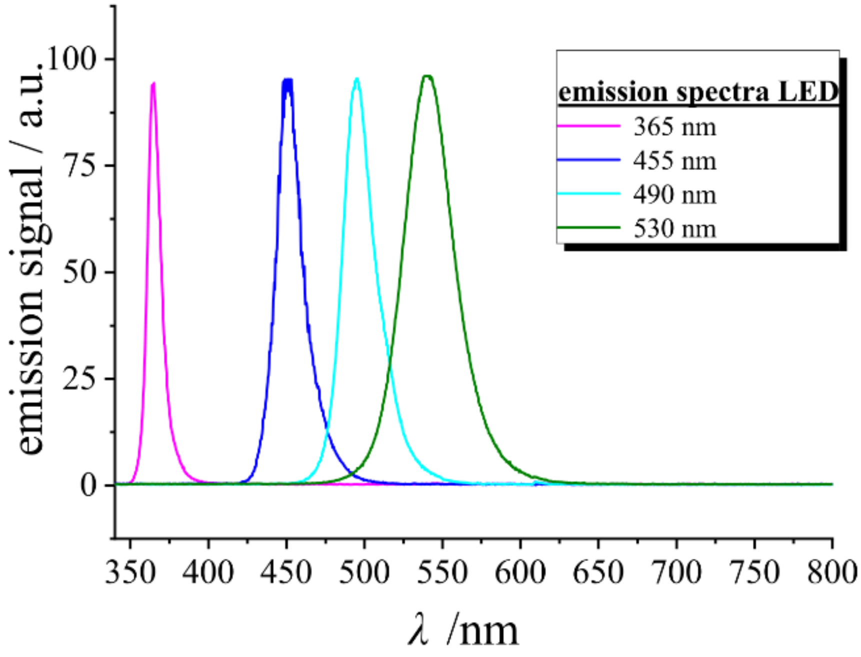

Figure S 25: Emission spectra of LED lamps used in this work.

3.1 Kinetic Investigations

Here supplementary discussions about the isomerization kinetics of compound **7**, both, under pH-neutral and acidic conditions are provided.

Figure S 26 shows the absorption characteristics in its thermally relaxed MC(H^+) form and after irradiation (Figure S 26a, c under neutral, Figure S 26b, d under acidic conditions). Figure S 26c and d depicts the corresponding time-evolution of the isomerization reaction as concluded from the absorption values of characteristic bands.

As the measurements are performed by a continuous irradiation with fixed wavelengths (neutral solution pH, λ = 540 nm; acidic solution pH, λ = 432 nm), the fraction %MC of the merocyanine could be calculated according to the following equation:

$$\%MC = \frac{Abs_{\mathrm{MC}}(t) - Abs_{\mathrm{SP}}}{Abs_{\mathrm{MC,max}} - Abs_{\mathrm{SP}}},$$

with the absorbance of merocyanine into the respective isomer mixture at the time point t $Abs_{MC}(t)$, the absorbance of pure spiropyrane isomer Abs_{SP} (assumed for neutral solution pH to be Abs_{SP} = 0 @ 540 nm, Figure S 26a, and for acidic solution pH to be Abs_{SP} = 0.37 @ 432 nm, Figure S 26b), $Abs_{MC,max}$ as the maximum MC isomer fraction (assumed for neutral solution pH to be $Abs_{MC,Max}$ = 1 @ 540 nm (Figure S 26a) , and for acidic solution pH to be $Abs_{MC,max}$ = 1.42 @ 432 nm; (Figure S 29b)).

The respective data is presented in Figure S 26c and d. The time-dependent Abs_{MC} (t) is, furthermore, fitted using a simple-exponential decay characterized by the decay time T_1 following

$$Abs_{\mathrm{MC}}\,(t) = \left(Abs_{\mathrm{MC,max}} - Abs_{\mathrm{SP}}\right) \cdot \exp\left(-\frac{t}{T_1}\right) + Abs_{\mathrm{SP}}.$$

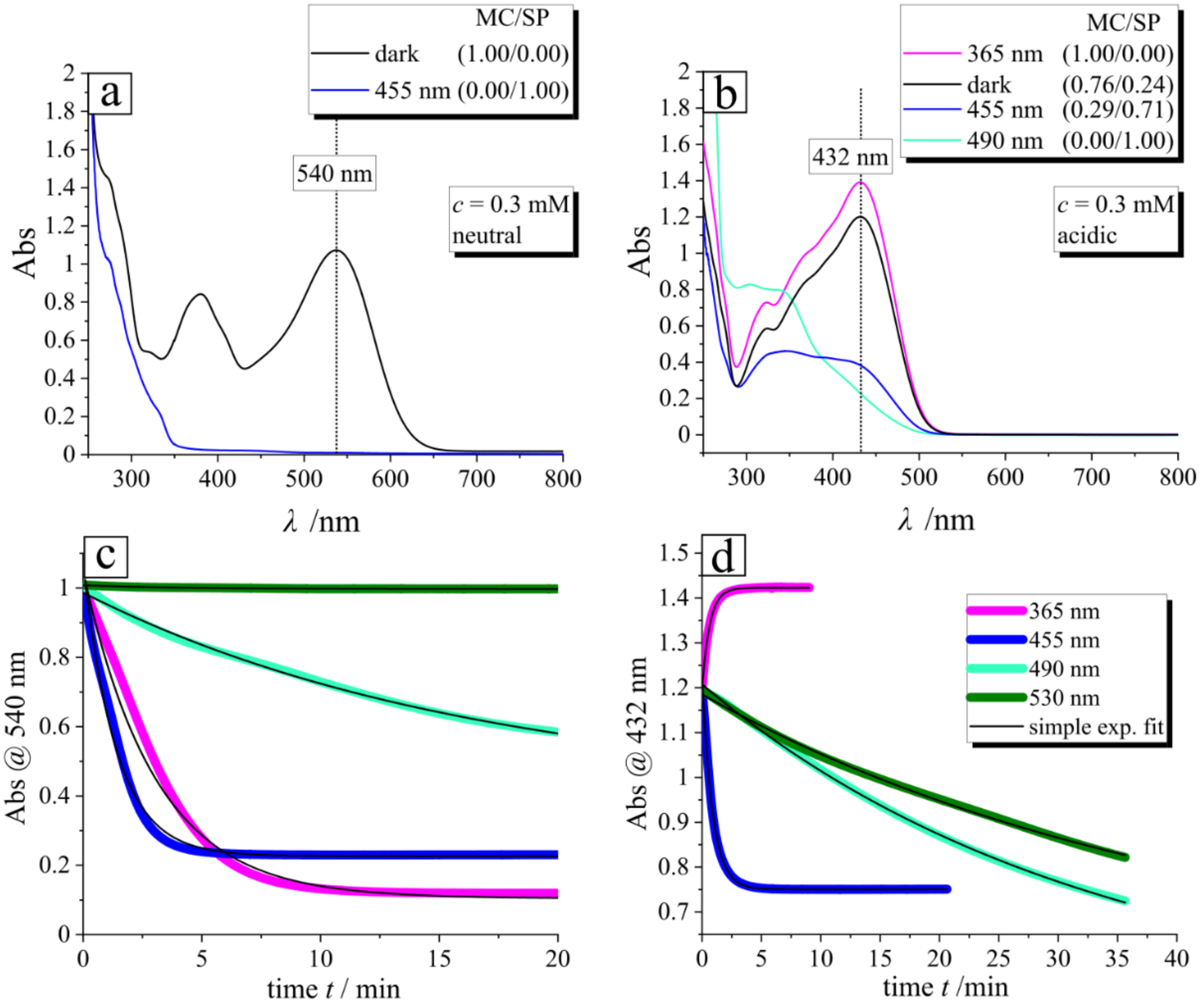

Model	ExpDec1			
Equation	y = A1*exp(-x/t1) + y0			
Plot	530 nm	365 nm	455 nm	490 nm
y0	1.00 ± 4.84E-6	0.10 ± 3.01E-4	0.23 ± 1.42E-4	0.41 ± 9.05E-4
A1	0.01 ± 1.08E-5	1.00 ± 9.81E-4	0.84 ± 9.13E-4	0.58 ± 8.10E-4
t1	4.09 ± 0.01	3.05 ± 0.01	1.40 ± 0.00	16.62 ± 0.04
Reduced Chi-Sqr	0.00	0.00	0.00	0.00
R-Square (COD)	0.99	0.99	0.99	1.00
Adj. R-Square	0.99	0.99	0.99	1.00

Model	ExpDec1			
Equation	y = A1*exp(-x/t1) + y0			
Plot	365 nm	455 nm	490 nm	530 nm
y0	1.42 ± 2.21E-5	0.75 ± 2.15E-5	0.47 ± 4.12E-4	0.56 ± 9.77E-4
A1	-0.23 ± 1.40E-4	0.47 ± 1.90E-4	0.73 ± 3.76E-4	0.62 ± 9.21E-4
t1	0.64 ± 6.19E-4	0.88 ± 5.21E-4	32.85 ± 0.03	41.78 ± 0.10
Reduced Chi-Sq	0.00	0.00	0.00	0.00
R-Square (COD	1.00	1.00	1.00	1.00
Adj. R-Square	1.00	1.00	1.00	1.00

Figure S 26: UV/Vis spectra of the surfactant **7** in MilliQ water with concentration of 0.3 mM for different wavelengths (a) neutral solution pH and (b) acidic solution pH. Dashed dotted line illustrate the monitoring wavelength. (c) absorbance (raw data) as a function of time for (c) neutral solution pH (d) acidic solution pH. Data was fitted with a simple exponential fit model. Corresponding fitting values are shown in table below the figure.

In a next step, the absorption intensities as concluded from Figure S 26 were translated into mole fractions of merocyanine MC(H^+) as shown in Figure S 27. The experimental data of MC (%*MC*) was fitted with a simple exponential fit function describing first order kinetic rate law:

$$\%MC\ (t) = \left(\%MC_0 - \%MC_{\mathrm{eq}}\right) \cdot \exp\left(-\frac{t}{T_1}\right) + \%MC_{\mathrm{eq}},$$

whereby %*MC* (t) represents mole fraction in dependency of time *t* with characteristic decay time T_1, $\%MC_0$ and $\%MC_{eq}$ as the mole fraction at initial time t_0 and in equilibrium.

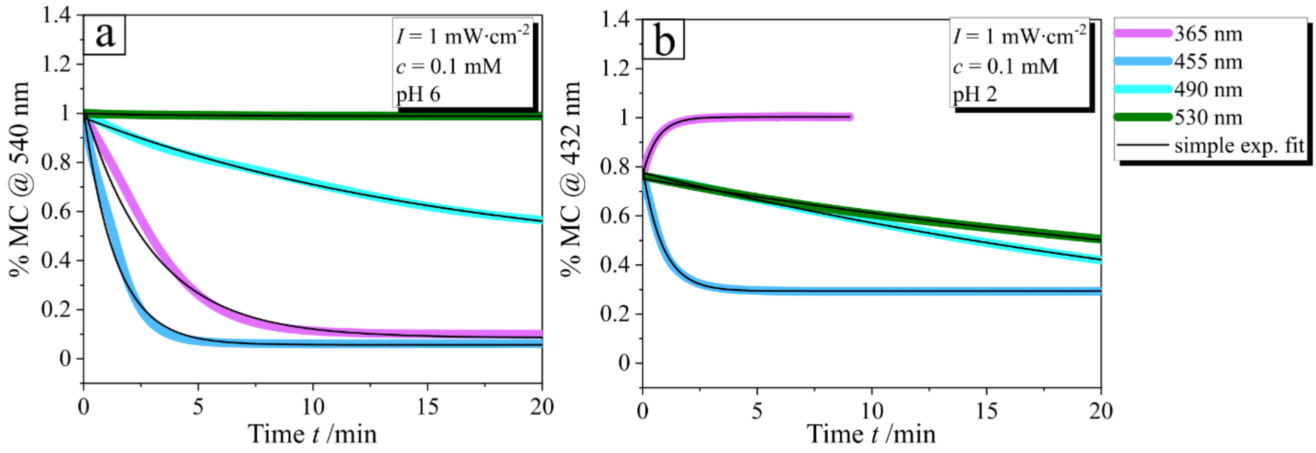

Figure S 27: Kinetic investigation of the switching process describing the conversion of the MC fraction of the surfactant to its SP form in MilliQ water. (a) neutral solution pH tracked with monitoring beam at 540 nm, (b) acidic solution (c = 0.5 M, 3 eq. of CF_3COOH) pH tracked with monitoring beam at 432 nm. Note, that the irradiation with 365 nm leads to increased absorption signals. Hence, this curve describes the temporal evolution of the MCH^+ form, which refers to the back reaction of the process.

The fitting parameters concluded from these curves reveal isomerization reaction half-lives as well as molar fractions of $MC(H^+)$ in dependence of the wavelengths of irradiation light, which are characteristic for the photosystem. Note, that the thermic relaxation results in a complete conversion of the SP to MC form, whereas photo-activation yields equilibria with the simultaneous presence of both forms. To provide a better overview, these parameters are summarized in Table S 1. The data reveal rapid conversions with reaction half-lives of approx. 6 min for the thermodynamic relaxation of the SP to the MC form as well as half-lives ranging from 1 to 16 min for the photochemically induced transfer from MC to SP depending on the wavelength (I = 1 mW cm^{-2}) used for irradiation.

Table S 1: Kinetic characterization of the switching process in MilliQ water and under acidic conditions. For illumination, LED lamps were used (emission spectra are shown in **Figure S 25**). Alongside the half-lives of the switching reaction, also molar fractions of the MC forms in the equilibria resulting from the illuminations are provided. These values indicate, that a photochemical treatment of the compounds often did not result in a complete conversion of one form to the other. The numbers in parenthesis represent the uncertainty of the last decimals of the measurement value.

			**$T_{1/2}$**	**Mole fraction of MC in the equilibrium**
**Neutral conditions**	SP → MC	26°C	6 min 12 s	1.0
		λ = 365 nm	3 min 2 s	0.0850 (2)
	MC → SP	λ = 455 nm	1 min 24 s	0.0568 (3)
		λ = 490 nm	16 min 37 s	0.380 (10)
		λ = 530 nm	4 min 5 s	0.988 (5)
**Acidic conditions**	SP → MC	26°C	37 min 24 s	0.78
		λ = 365 nm	38 s	1.003 (23)
	MC → SP	λ = 455 nm	53 s	0.293 (22)
		λ = 490 nm	32 min 51 s	0.000 (4)
		λ = 530 nm	41 min 47 s	0. 093 (9)

All intensities were acquired using LED light with I = 1 mW cm^{-2}.

To investigate the kinetic behavior of the relaxation reaction in more detail, the relaxation (after switching into the SP form) was investigated in dependence of the temperature. Figure S 27 reveals the absorption at characteristic bands as a function of time (a and b), with c depicting the characteristic half-lives as a function of temperature and d showing an Arrhenius plot.

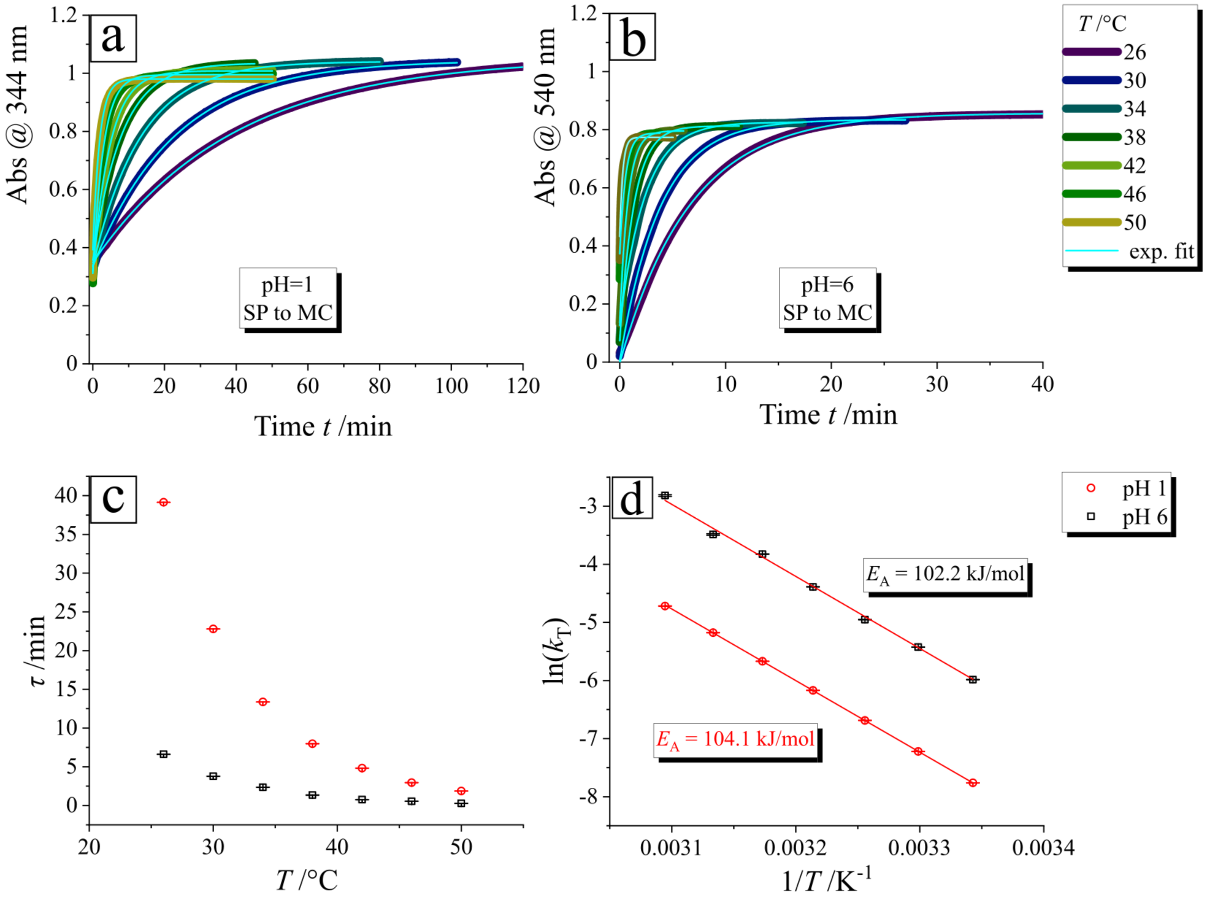

Figure S 28: Kinetic investigation of the switching process describing the thermic relaxation process of the SP form of the molecule to the MC form under different temperatures in (a) acidic water (c = 0.5 M, 3 eq. of CF_3COOH) and (b) neutral water. (c) decay time as a function of temperature, (d) Arrhenius plotting style of data presented in (c).

3.2 Determination of the pH-dependent behavior of the surfactant

In this section, the characterization of surfactant 7 regarding its protonation behavior in aqueous solution, depending on its switching state, is discussed.

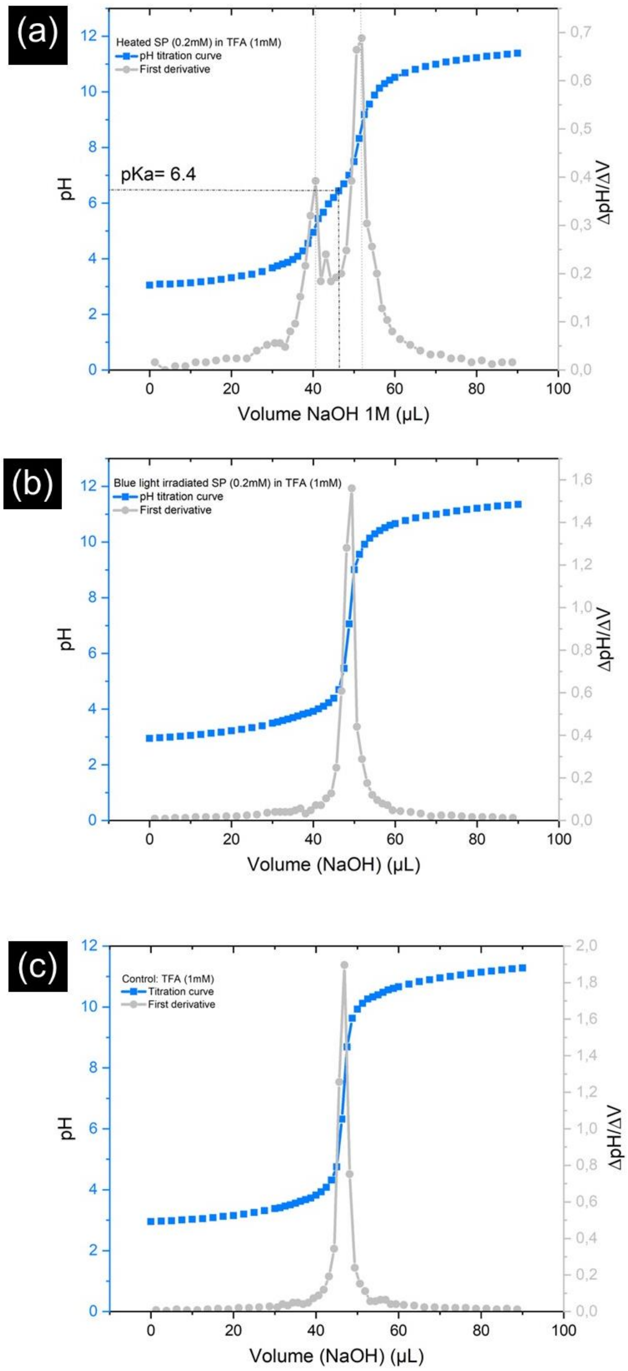

Figure S 29: Potentiometric titration curves of the surfactant under (a) thermally relaxed conditions, (b) photoirradiation with blue light. For titration, the sample was first treated with CF_3COOH and afterwards subjected to a back titration with NaOH. (c) represents the curve of the blind value, *i.e.* the titration curve of CF_3COOH with NaOH. For better visualization of inflection points, the first derivatives of curve (a) and (b) are displayed. The curves exhibit an inflection point at pH = 6.4 for (a), but not for (b), which mean that a pK_a value for the reaction MC -> MCH^+ can be determined with 6.4.

Accordingly, the titration curve reveals a protonation of the molecule in its thermally relaxed form at a pH value of 4.5, being indicative for the protonation of MC to MCH^+, while the absence of an inflection point in the titration curve of the molecule after irradiation with blue hints towards the fact that SP form does not undergo a protonation reaction in the monitored pH range. Figure S31 shows a set of the respective UV/Vis absorption spectra under different pH values.

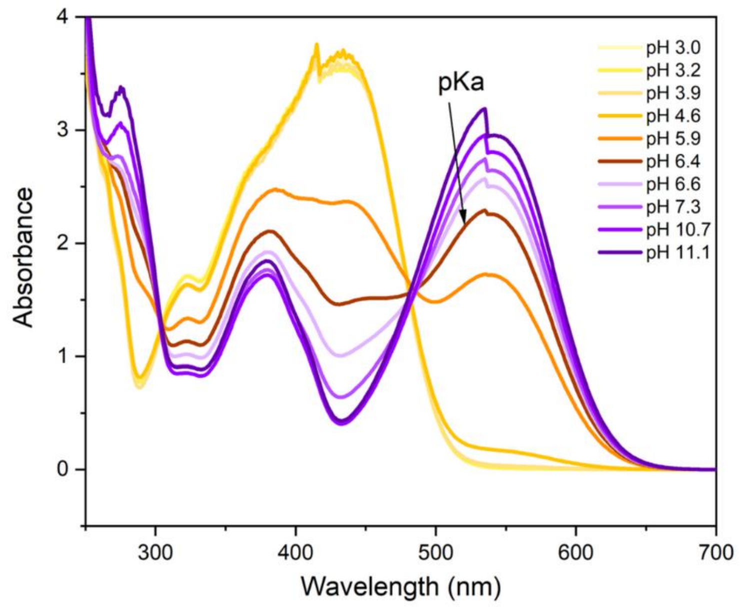

Figure S 30: Absorption spectra of the surfactant under thermally relaxed conditions at different pH values.

4. Micellization behavior of the surfactant

Here, supplementary insights into the micellization behavior of the surfactant are provided.

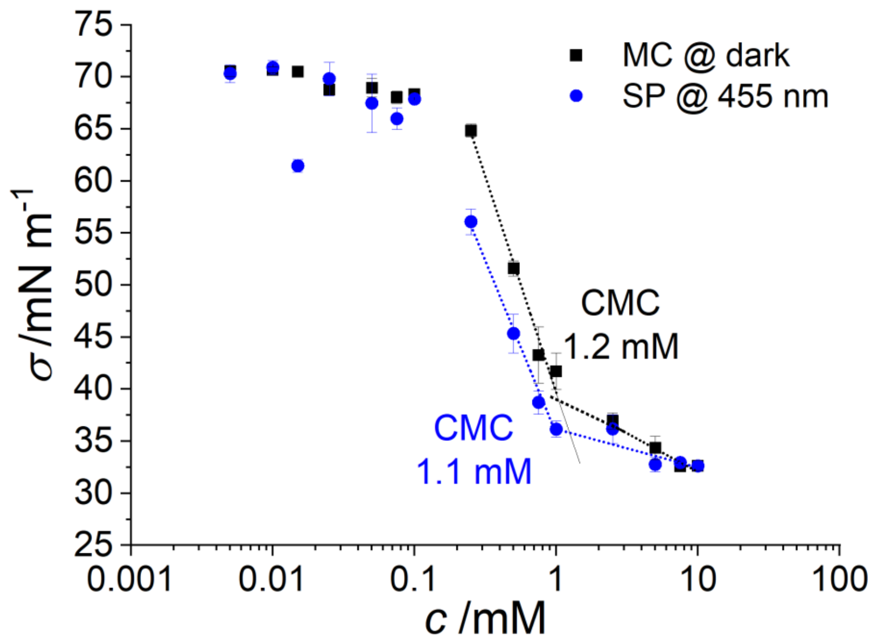

Figure S 31: CMC determination of compound **7** in MilliQ water. Note, that the determined CMCs differ in their absolute value, but not notably in their relative values to each other. We explain the reduced CMCs under alkaline conditions by the increased ionic strengths in the aqueous solution therein.

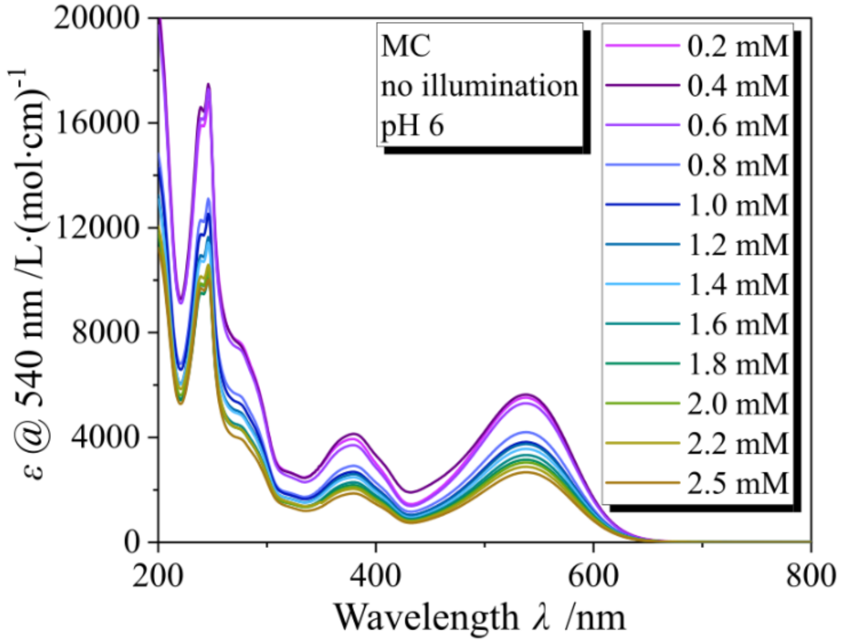

Figure S 32: UV/Vis spectra of the surfactant **7** in MilliQ water under different concentrations. To obtain an improved insight into possible self-assembly processes, the molar extinction coefficient ε is plotted against the absorption wavelength. In a solution, in which the molecules do not show an intermolecular interaction, ε is supposed to be independent of the concentration. In the spectra shown here, however, a decrease of characteristic molar extinction coefficients is obvious for concentrations > 0.8 mM. In solutions with concentrations higher than this (conc. > CMC of the system), self-assembly processes occur. Since these processes are accompanied by a re-arrangement of the delocalized π-system of the molecule, namely by its conversion from the MC to the SP form, species absorbing at the wavelengths characteristic for MC disappear from the solution. This is reflected by decreased ε.

Explanations on the supplementary video V1:

Diluting a surfactant solution with a high concentration (containing a mixture of MC and micellized SP molecules) to a lower concentration changes the intensity of the red color. Interestingly, a slightly red-colored solution intensifies during the dilution process. This can be explained by the fact that during the addition of water, remaining SP molecules forming micelles of are now expelled into the bulk solution to form molecules in the MC form therein (corresponding to an equilibrium shift of aggregated SP molecules due to dilution). The thermal relaxation switches over time all single SP molecules back to the MC state and the red intense color appears again over the time. The observations from Video S1 can, therefore, fairly be explained.

5. Theoretical considerations

Having discussed the thermodynamic details of compound **7** based on quantum-chemical calculations (Figure 4 in the main part of the manuscript), we here want to provide additional insights.

Note, that we occasionally label *open E* forms (MC) with *ctc, ttc,* and *ttt.* The three letters refer to the configuration of three dihedral angles, NCCC, CCCC, and CCCC(O), whereby *c* labels the *cis,* and *t* the *trans* configuration.

Figure S 34 schematically shows a reaction path between the lowest energy closed and open forms, *Closed 1* and *Open E ttt* (named I and IV, respectively, in Figure 4 of the main text). Gibbs free energies (relative to the *Closed 1* form) of minima and TSs are indicated.

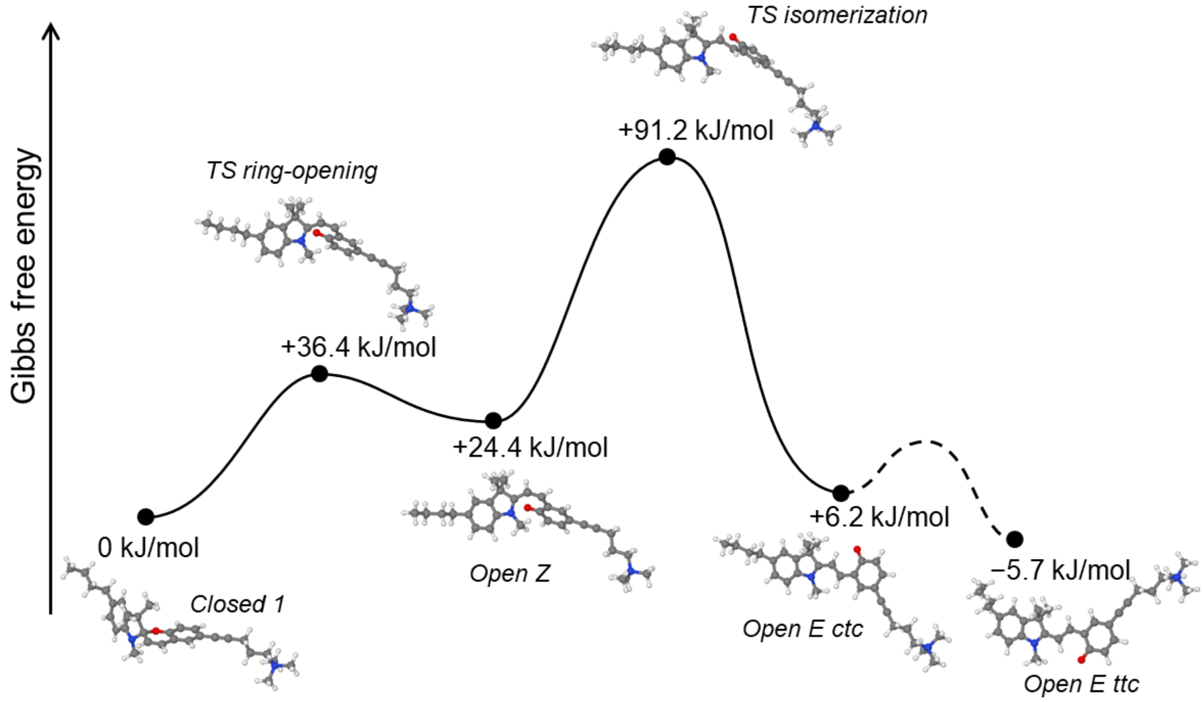

Figure S 33: SP→MC switching reaction scheme (starting from the Closed 1 form, named I in Fig.4 of the main text) in the electronic ground state. Critical points were located at the B3LYP+D3(BJ)/def2-TZVP/PCM(water) level. The relative Gibbs free energies (calculated at the same level) are shown. For comparison, the experimentally determined activation energy is 102 kJ mol^{-1} (Figure S 26), which is in fair agreement with the calculated value. Note, that the energy barrier is in good agreement with the activation energy as determined in Figure S 28.

Figure S 35 shows relative Gibbs free energies and corresponding structures of the *protonated* closed (SPH^+) and open (MCH^+) forms of the surfactant. Again, several minima were found (see structures I-IV in Figure S34). In particular, open forms I and II differ by rotation of oxygen-containing ring around the adjacent CC bond, whereas closed forms III and IV differ in spatial arrangement of the additional proton and the nearby methyl group with respect to the ring system as shown in Figure S35. We note that here protonation is assumed to occur on oxygen for the open form and on nitrogen of spiropyran for the closed form. The SPH^+ form is ~ +70 kJ mol^{-1} higher in Gibbs free energy than the MCH^+ form. Protonation of SP on oxygen resulted in ring-opening during geometry optimization, in agreement with an earlier computational report for another spiropyran derivative.[11]

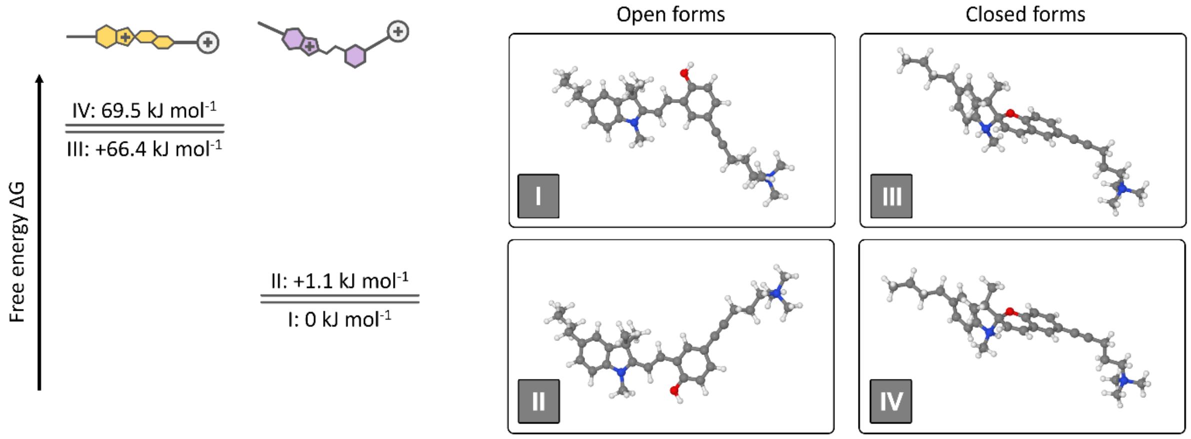

Figure S 34: Protonated closed (SPH^+) and open (MCH^+) forms of the surfactant with the corresponding relative Gibbs free energies (with respect to the Open E ttt form, named I in this figure) calculated at the B3LYP+D3(BJ)/def2-TZVP/PCM (water) level.

Furthermore, we have estimated the reaction Gibbs free energy for the following hypothetical reactions:

$$SP + H_3O^+ \rightleftharpoons SPH^+ + H_2O \quad (\alpha)$$

and

$$SP + H_3O^+ \rightleftharpoons MCH^+ + H_2O \quad (\beta).$$

In case of reaction (α) we consider protonation on the N atom, whereas for reaction (β) on the O atom. Using B3LYP+D3(BJ)/def2-TZVP/PCM (water), we obtain for reaction (α):

$$\Delta G = G(SPH^+) + G(H_2O) - [G(SP) + G(H_3O^+)] \approx -106 \text{ kJ mol}^{-1}$$

and for reaction (β):

$$\Delta G = G(MCH^+) + G(H_2O) - [G(SP) + G(H_3O^+)] \approx -172 \text{ kJ mol}^{-1} .$$

Thus, MCH^+ is much more stable than SP at acidic conditions. Finally, we note that spiropyrans were reported to undergo a spontaneous ring-opening upon protonation.[11]

6. Emulsification experiments

Here, the emulsification experiments are discussed in more detail. Figure S 36 shows light micrographs of emulsions, which are displayed in Figure 6 in the main part of the manuscript.

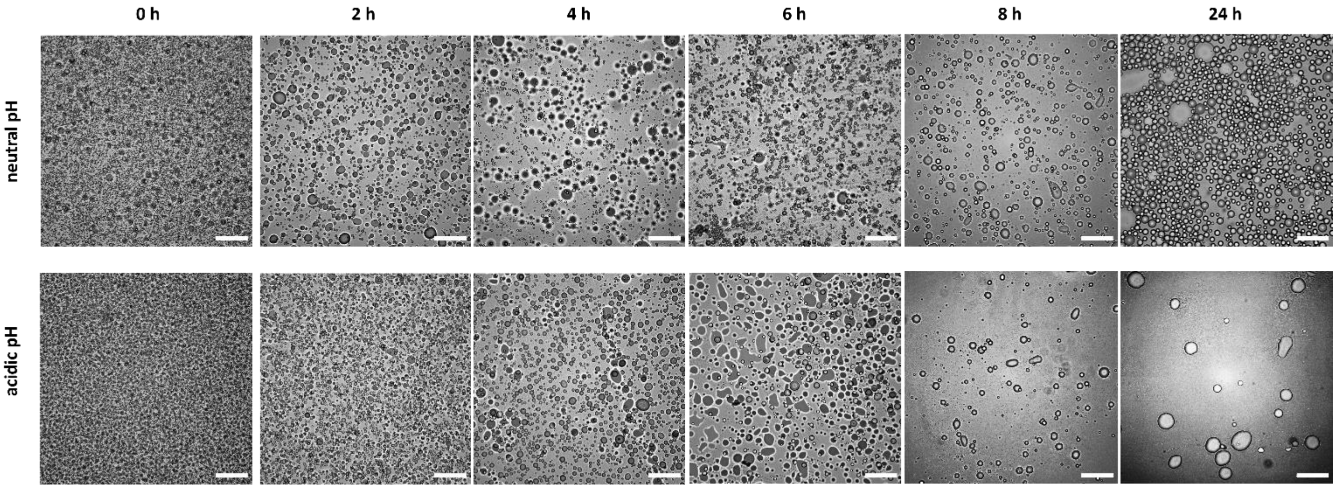

Figure S 35: Light microscopy images of the emulsions shown in Figure 5 in the manuscript. During 6 to 8 h, the emulsions possess a reasonable stability. After one day, however, the integrity of the acidic sample is severely affected. The scalebars are 100 µm.

Based on the pH value and the GC/MS conversion, the reaction progress can be monitored. Figure S 36 shows the respective GC/MS data.

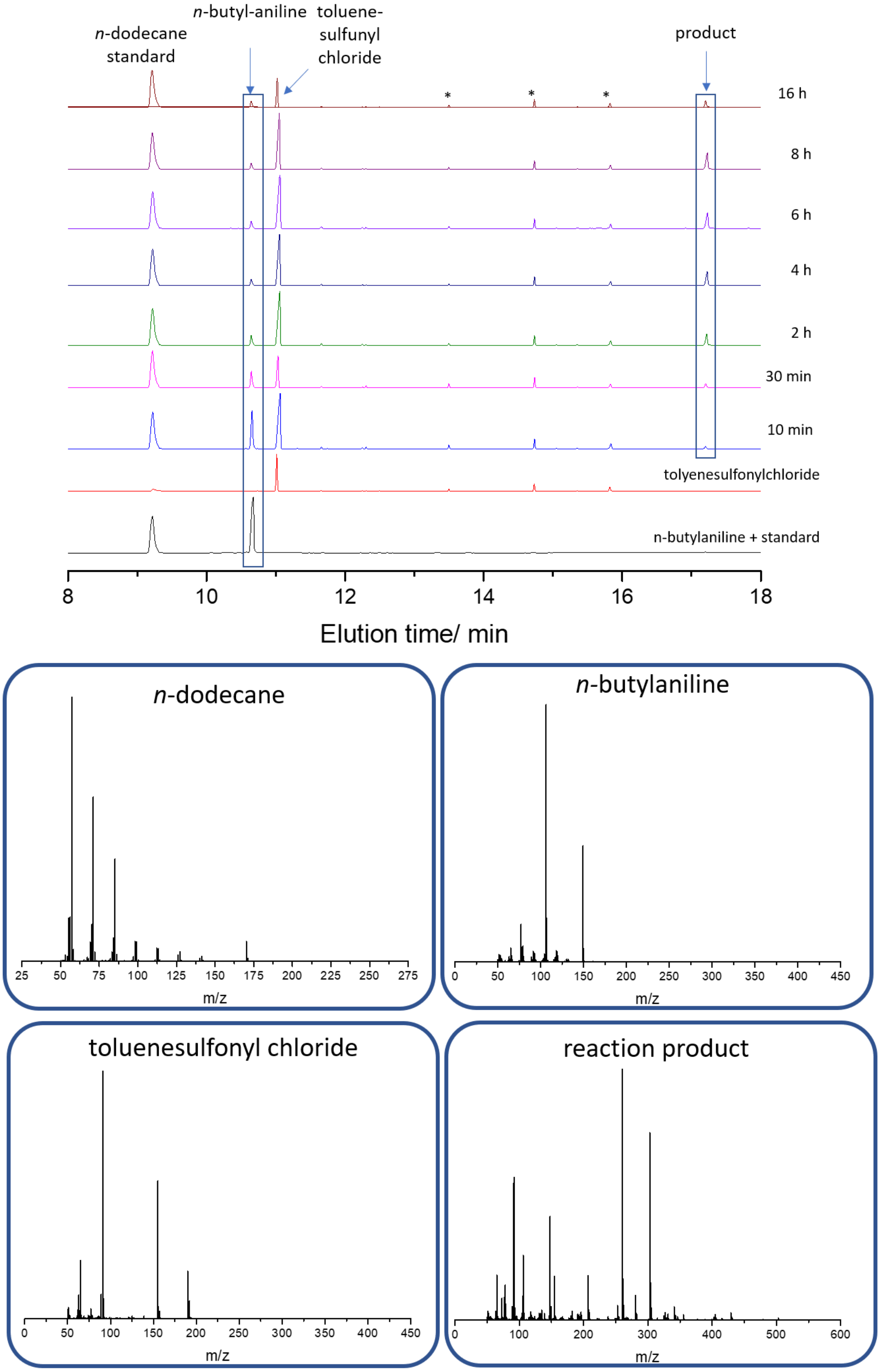

Figure S 36: Gas chromatographs of the emulsions used for the reaction control. Top: Elugrams after different reaction times. The signals indicated with (*) correspond to impurities, which are contained in the toluenesulfonyl chloride batch. Bottom: Mass spectra of characteristic peaks in the chromatogram.

As a comparison experiment to the one shown in Figure 7 in the manuscript, an emulsion was prepared, which merely contains toluene sulfonyl chloride (emulsion (b), see section 1.6.4 in the SI). As this compound rapidly hydrolyses, the emulsion can be destabilized already after two hours.

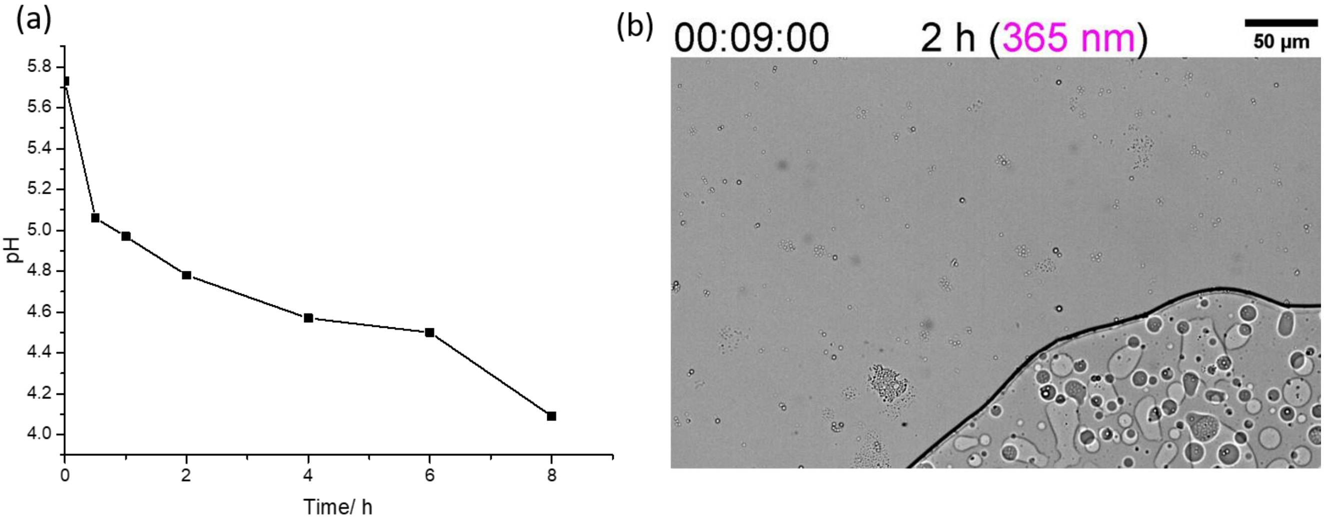

Figure S 37: Behavior of an emulsion, where *p*-toluenesulfonyl chloride is dissolved in the toluene phase. (a) pH dependency. As a matter of the hydrolysis reaction, the emulsion quickly acidifies. (b) Image of an emulsion, which has been irradiated for 9 min with UV light, sampled after 2 h. Due to the rapid pH decrease as a result of the quick hydrolysis reaction, the surfactant-stabilized emulsion can be destabilized with UV light.

References:

[1] Gaussian 16, Revision B.01, Frisch, M.J., Trucks, G.W., Schlegel, H.B., Scuseria, G.E., Robb, M.A., Cheeseman, J.R.; Scalmani, G.; Barone, V.; Petersson, G.A.; Nakatsuji, H.; Li, X.; Caricato, M.; Marenich, A.V.; Bloino, J., Janesko, B.G., Gomperts, R., Mennucci, B., Hratchian, H.P., Ortiz, J.V., Izmaylov, A.F., Sonnenberg, J.L., Williams-Young, D., Ding, F., Lipparini, F., Egidi, F., Goings, J., Peng, B., Petrone, A., Henderson, T., Ranasinghe, D., Zakrzewski, V.G., Gao, J., Rega, N., Zheng, G., Liang, W., Hada, M., Ehara, M., Toyota, K., Fukuda, R., Hasegawa, J., Ishida, M., Nakajima, T., Honda, Y., Kitao, O., Nakai, H., Vreven, T., Throssell, K., Montgomery Jr., J.A., Peralta, J.E., Ogliaro, F., Bearpark, M.J., Heyd, J.J., Brothers, E.N., Kudin, K.N., Staroverov, V.N., Keith, T.A., Kobayashi, R., Normand, J., Raghavachari, K., Rendell, A.P., Burant, J.C., Iyengar, S.S., Tomasi, J., Cossi, M., Millam, J.M., Klene, M., Adamo, C., Cammi, R., Ochterski, J.W., Martin, R.L., Morokuma, K., Farkas, O., Foresman, J.B., Fox, D.J. Gaussian, Inc., Wallingford CT (2016).
[2] A. D. Becke, *J. Chem. Phys.* **1993**, *98*, 5648–5652.
[3] P. J. Stephens, F. J. Devlin, C. F. Chabalowski, M. J. Frisch, *J. Phys. Chem.* **1994**, *98*, 11623–11627.
[4] F. Weigend, R. Ahlrichs, *Phys. Chem. Chem. Phys.* **2005**, *7*, 3297–3305.
[5] S. Grimme, S. Ehrlich, L. Goerigk, *J. Comput. Chem.* **2011**, *32*, 1456–1465.
[6] S. Miertuš, E. Scrocco, J. Tomasi, *Chem. Phys.* **1981**, *55*, 117–129.
[7] G. Scalmani, M. J. Frisch, *J. Chem. Phys.* **2010**, *132*, 114110.
[8] K. Fukui, *Acc. Chem. Res.* **1981**, *14*, 363–368.
[9] H. P. Hratchian, H. B. Schlegel, in *Theory and Applications of Computational Chemistry* (Eds.: C.E. Dykstra, G. Frenking, K.S. Kim, G.E. Scuseria), Elsevier, Amsterdam, **2005**, pp. 195–249.
[10] P. C. Hariharan, J. A. Pople, *Theoret. Chim. Acta* **1973**, *28*, 213–222.
[11] L. Kortekaas, J. Chen, D. Jacquemin, W. R. Browne, *J. Phys. Chem. B* **2018**, *122*, 6423–6430.
